# The Clinical Significance of DC-SIGN and DC-SIGNR, which Are Novel Markers Expressed in Human Colon Cancer

**DOI:** 10.1371/journal.pone.0114748

**Published:** 2014-12-12

**Authors:** Yanmei Jiang, Changfu Zhang, Kai Chen, Zhe Chen, Zhigang Sun, Zhuqing Zhang, Dongbing Ding, Shuangyi Ren, Yunfei Zuo

**Affiliations:** 1 Department of Clinical Biochemistry, College of Laboratory Diagnostic Medicine, Dalian Medical University, Dalian, 116044, China; 2 Department of Surgery, the Second Affiliated Hospital of Dalian Medical University, Dalian, 116023, China; 3 Department of Clinical Laboratory, the First Affiliated Hospital of Dalian Medical University, Dalian, 116011, China; 4 Department of Neurosurgery, the First Affiliated Hospital of Dalian Medical University, Dalian, 116011, China; The University of Hong Kong, China

## Abstract

**Background:**

Colon cancer has always been diagnosed at a late stage, which is associated with poor prognosis. The currently used serum tumor markers CEA and CA19-9 display low sensitivity and specificity and may not have diagnostic value in early stage colon cancer. Thus, there is an urgent need to identify novel serum biomarkers for use in the early detection of colon cancer.

**Methods:**

In this study, the expression of DC-SIGN and DC-SIGNR in serum was detected by enzyme-linked immunosorbent assay (ELISA). DC-SIGN and DC-SIGNR expression was detected in cancer tissues by immunohistochemistry (IHC).

**Results:**

The level of sDC-SIGN was lower in patients than in the healthy controls, while the level of sDC-SIGNR in patients was higher than in the healthy controls. Both sDC-SIGN and sDC-SIGNR had diagnostic significances for cancer patients, and the combined diagnosis of these two markers was higher than both of them alone. Furthermore, there were significant differences between both sDC-SIGN and sDC-SIGNR in stage I/II patients and the healthy controls. Moreover, high sDC-SIGN level was accompanied with the long survival time. Additionally, DC-SIGNR was negative in the cancer foci and matched normal colon tissues but was weakly positive between the cancer foci. DC-SIGN staining was faint in matched normal colon tissues, strong in the tumor stroma and the invasive margin of colon cancer tissues, and negatively correlated with the sDC-SIGN level in serum from the same patient. Interestingly, the percent survival of patients with a DC-SIGN mean density of>0.001219 (the upper 95% confidence interval of matched normal colon tissues) was higher than for all other patients.

**Conclusion:**

DC-SIGN and DC-SIGNR are blood-based molecular markers that can potentially be used for the diagnosis of early stage patients. Moreover, expression of DC-SIGN in serum and cancer tissues may affect the survival time for colon cancer patients.

## Introduction

There were an estimated 3.45 million new cases of cancer (excluding non-melanoma skin cancer) and 1.75 million deaths from cancer in Europe in 2012 [1], resulting in the second highest incidence and mortality rates worldwide. Colorectal cancer (CRC) is the most common gastrointestinal cancer worldwide, with the incidence of colon cancer increasing in most countries over the past 20 years [2]. Colon cancer is often diagnosed at an advanced stage, leading to a poor prognosis [3]–[6]. As the current clinical procedures utilized for disease diagnosis are invasive, unpleasant, and inconvenient, the development of simple blood tests that can be used for early detection would be beneficial for ultimately controlling and preventing CRC [3], [5]–[6]. Serum tumor markers, such as Carcinoembryonic antigen (CEA) and Carbohydrate antigen 199 (CA19-9), greatly improve diagnosis. However, their application is limited to surveillance postsurgery, and they are not suitable for the early detection of colon cancer, as their sensitivity and specificity are very low [7]–[9]. Therefore, there is a need for novel early colon tumor markers.

Recently, it has become apparent that C-type lectins play an important role in tumor prognosis. Caligaris-Cappio and colleagues have reported that the expression of CD23 and plasma sCD23 was most likely to have diagnostic and prognostic significance in B cell chronic lymphocytic leukemia (B-CLL) [10]–[11]. Ferroni and colleagues found that for pre-surgical CRC patients, serum levels of sE-selectin were correlated with overall prognosis and could potentially guide treatment [12]. Moreover, we previously reported that LSECtin (liver and lymph node sinusoidal endothelial cell C-type lectin) played an important role in colorectal carcinoma liver metastasis and may be a promising new target for intervention in metastasis formation [13]. Importantly, the dendritic cell-specific ICAM-3 grabbing nonintegrin (DC-SIGN) dependent interaction of immature dendritic cells (DCs) with some colorectal carcinoma cells may suppress DC functional maturation, inducing the failure of the host to initiate a powerful antitumor response [14]–[15].

The membrane-bound C type lectins, DC-SIGN and its homologue DC-SIGNR (DC-SIGN-related protein, also known as L-SIGN, CD209L) are located on human chromosome 19p13.3 and belong to a subfamily in the lectin gene cluster along with the above-mentioned CD23 and LSECtin [16]–[17]. DC-SIGN presents on the surface of mature DCs in the lymph node as well as immature monocyte-derived and interstitial DCs in the placenta, cervical mucosa, uterus and colon [18]–[20]. In contrast, DC-SIGNR is found on endothelial cells in the placenta, liver and lymph nodes [21]. Although DC-SIGNR is 77% identical to DC-SIGN according to the amino-acid sequence [22], the relationship between DC-SIGNR and colon cancer has not been reported. However, our team previously reported that the level of DC-SIGNR expression in serum was low in Non-Hodgkin lymphoma (NHL) and may have potential use in the clinical setting [23].

In the present study, we identified soluble DC-SIGN (sDC-SIGN) and DC-SIGNR (sDC-SIGNR) in serum from colon cancer patients. sDC-SIGN and sDC-SIGNR showed significant potential as novel markers for the diagnosis of colon cancer in early stage patients. Moreover, the level of sDC-SIGN may have prognostic significances for cancer patients. Additionally, the expression of DC-SIGN and DC-SIGNR was detected in colon cancer tissues, and the level of DC-SIGN expression in cancer tissues can be used as an indicator of disease prognosis.

## Materials and Methods

### Reagents and Antibodies

Recombinant human DC-SIGN IgG-Fc fusion protein (rhDC-SIGN-Fc), DC-SIGNR IgG-Fc (rhDC-SIGNR-Fc) and anti-DC-SIGNR mouse monoclonal antibody (detection antibody) were purchased from R&D Systems (Minneapolis, MN, USA). Monoclonal anti-human DC-SIGN (capture antibody) produced in mouse was purchased from SIGMA-ALDRICH, INC, USA. A rabbit polyclonal antibody to DC-SIGN (detection antibody) and a monoclonal DC-SIGNR antibody (capture antibody) were purchased from Abcam, INC, Hong Kong, China. Another rabbit monoclonal antibody to DC-SIGNR was purchased from Epitomics, Hong Kong, China. Horseradish peroxidase (HRP) conjugated goat-anti-rabbit and anti-mouse antibodies, goat serum blocking reagent and 3,3′-diaminobenzidine tetrahydrochloride (DAB) were obtained from ZSGB-BIO (Beijing, China), and 3,3′,5,5′-tetramethylbenzidine (TMB) was purchased from TIANGEN BIOTECH CO, LTD, Beijing, China.

### Clinical samples

We obtained the oral informed consent by participants or the next of deceased patients for their clinical records to be used in this study. And patients or next of kin also provided oral consent for the use of their tissue and serum samples in this study. These deceased samples were obtained in an anonymized form. We record the participants consent by the list of their names, and the patients or the next of the deceased patients agreed with it. Meanwhile, the Dalian Medical University research ethics committee approved this consent procedure. The protocols and procedures were approved by the Dalian Medical University research ethics committee and were based on the guiding policy and mechanism, and informed consent was obtained from all participants. Serum samples were collected from 182 patients who had been diagnosed with colon cancer by pathological examinations, either during surgical operations or colonoscopy, during the period from 2011 to 2013 at the First or Second Affiliated Hospital of Dalian Medical University and were stored at -80°C until they were analyzed. None of the patients were directly related. The patient group consisted of 101 males and 81 females, with ages ranging from 23 to 86 years (mean: 61). The disease stage for the patients was confirmed according to cancer staging criteria of the 7th edition staging American Joint Committee on Cancer (AJCC) [24]. The patients' details included gender, age, stage, tumor differentiation, and serum CEA, CA19-9 and survival time are shown in S1 Table and S2 Table. The control group was composed of 101 healthy blood donor volunteers (45 males and 56 females). They were chosen from routine health examinations based on the following selection criteria: all physical indicators were in the normal range and the volunteers were free of cancer, hepatitis, or infection, among others. Their ages ranged from 21 to 62 years.

In addition, 98 formalin-fixed paraffin-embedded colon cancer tissue samples (49 of which were from the colon cancer patients who had died post-operatively within the last eleven years), 4 lymph nodes and 30 matched normal colon tissues from patients from 2002 to 2013 were obtained from The First Affiliated Hospital of Dalian Medical University. Normal lymph nodes were used as either positive or negative controls, and matched normal colon tissues was used as a control group. The clinical data from these colon cancer patients is shown in S3 Table and S4 Table.

### sDC-SIGN and sDC-SIGNR in human serum were detected by standard sandwich ELISA

Ninety-six-well microplates were coated with 100 µl of capture antibody at a final concentration of 1 µg/ml in Na_2_CO_3_ buffer (pH 9.6), and the plate was covered with an adhesive plastic and incubated overnight at 4°C and subsequently washed three times with a phosphate buffered solution (PBS) containing 0.05% Tween-20 (PBST, PH 7.4). Then, the wells were blocked by adding 200 µl of blocking buffer (5% non fat dry milk/PBS) per well and incubating at 37°C for 90 min. After the plates had been washed, 100 µl of diluted rhDC-SIGN-Fc or rhDC-SIGNR-Fc standards (specific concentrations are shown in S1 Figure) were added to the wells in duplicate along with serum samples from patients and healthy individuals, and the plates were incubated at 37°C for 90 min. One hundred microliters of PBS was used as a negative control. Subsequently, the plates were washed three times with PBST, and 100 µl of a detection antibody diluted to a concentration of 1 µg/ml was added, followed by incubation at 37°C for 90 min. After washing, 100 µl of a horseradish peroxidase-conjugated goat-anti-rabbit antibody was added, and the plates were incubated for 60 min at 37°C, followed by washing three times. Finally, 100 µl of TMB (3,3′,5,5′-tetramethylbenzidine) was added to each well, followed by an incubation at 37°C for 30 min. The reaction was stopped by adding 2 mol/L H_2_SO_4_, and the optical density (OD) value was measured at 450 nm. Standard curve fitting was performed using CurveExpert 1.3 from serial dilutions, with the rhDC-SIGN-Fc or rhDC-SIGNR-Fc concentration on the Y axis vs the OD value on the X axis. The equation and the data are shown on S1A and S1B Figure, R^2^ = 0.9987 and R^2^ = 0.9971. The sDC-SIGN and sDC-SIGNR levels in patients (and the healthy controls) were read from the standard curve and are shown in S1 Table and S2 Table. To analyze the diagnostic value of sDC-SIGN and sDC-SIGNR, the ROC curves of sDC-SIGN and sDC-SIGNR were drawn using GraphPad Prism 5.

### Immunohistochemistry (IHC) (DC-SIGN detection as an example)

Before being deparaffinized in xylene and rehydrated in a graded ethanol series, sections from paraffin-embedded blocks were incubated at 60°C for 30 minutes. Endogenous peroxidase activity was quenched by incubation for 10 minutes in 3% hydrogen peroxide. Antigen retrieval was performed by microwaving for 15 minutes in a 0.01 M citrate-buffered solution, pH 6.0. Tissues were washed with PBS prior to incubation with goat serum for blocking. Sections were incubated with an anti-DC-SIGN rabbit pAb overnight at 4°C (1∶50). The next day, after being washed with PBS, the sections were incubated with horseradish peroxidase-labeled anti-rabbit immunoglobulin (1∶100) and were then washed again. Finally, the sections were developed using DAB for detection and were then counterstained with hematoxylin before observation under a light microscope. Morphometry: DC-SIGN immunostaining in colon cancer was assessed by (IOD sum)/Area using the Image Pro Plus image analysis software system. IOD (integrated optical density) sum represents the protein content of DC-SIGN in the area of interest (AOI), while Area equals the area of AOI. (IOD sum)/area stands for “mean density”. Briefly, images were captured at 200x magnification from 3 AOIs/case, which were selected based on areas with maximal DC-SIGN staining. Following image capture, DC-SIGN-staining positive areas were selected according to image Histogram Based (H: 0–29; S: 0–255; I: 0–230) within the AOI, and the “IOD sum” value was determined. Additionally, the AOI was analyzed based on another parameter (H: 0–255; S: 0–255; I: 0–230), and the “area” value was determined. The mean density values are shown in S3 Table and S4 Table. This quantitation was positively correlated with DC-SIGN expression in tissue.

### Statistical analysis

All of the data are expressed as the mean ± SD. The statistical significance among more than two groups was determined using the Kruskal-Wallis nonparametric test. The correlation of DC-SIGN and DC-SIGNR values with clinical parameters was tested by the non-parametric Spearman rank correlation coefficient test. In all of the tests, two-sided P values below 0.05 were considered significant. All statistical analyses and figures were performed using GraphPad Prism5 (Graphpad Software, Inc., San Diego, CA).

## Results

### The levels and diagnostic values of sDC-SIGN and sDC-SIGNR in colon cancer patients

**Figure 1:**
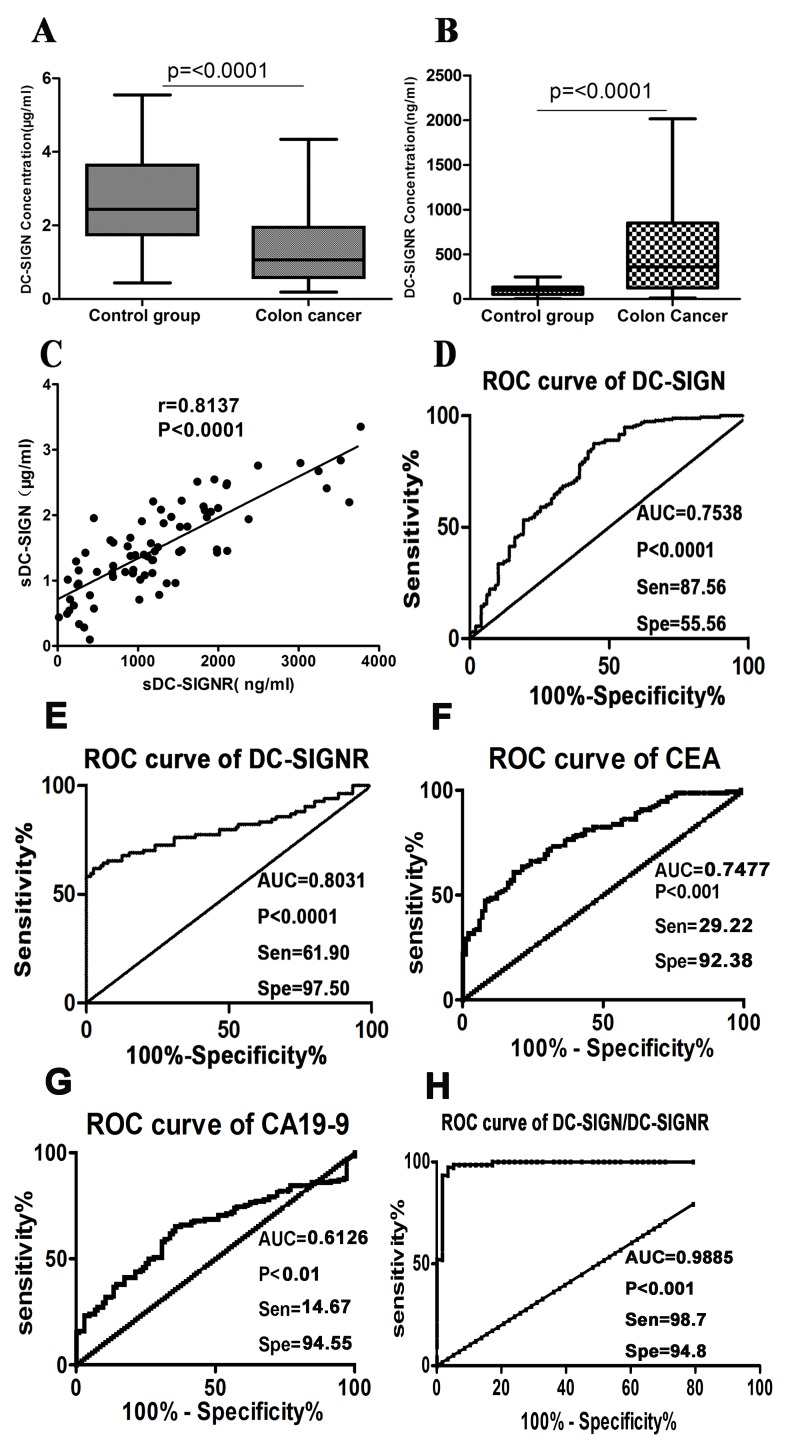
The levels and diagnostic values of sDC-SIGN and sDC-SIGNR in colon cancer patients. **A–B**: There was statistical significance in the sDC-SIGN and sDC-SIGNR level between healthy controls and colon cancer patients, P<0.001. The sDC-SIGN level (A) was lower in colon cancer patients than healthy controls; however, the sDC-SIGNR level (B) was higher in colon cancer patients than healthy controls. **C**: The level of sDC-SIGN in serum was significantly correlated with that of sDC-SIGNR in the same patient, P<0.001, r = 0.8137. **D–E**: According to the analysis of Youden index, the cut-off concentrations for sDC-SIGN and sDC-SIGNR are less than 2.226 µg/ml and more than 227.2 ng/ml, respectively, and the corresponding Sensitivity and Specificity of sDC-SIGN and sDC-SIGNR are 87.56%, 55.56% and 61.90%, 97.50%, respectively. Additionally, the Area under the curve (AUC) for sDC-SIGN is 0.7538, while the AUC of sDC-SIGNR is 0.8031. **F–G**: The AUC of CEA and CA19-9 in patients were 0.7477 and 0.6126, respectively. Based on the clinical decisive levels (0–5 ug/l for CEA, 0–27 U/ml for CA19-9), the cut-off values of CEA and CA19-9 were obtained. And the corresponding sensitivity and specificity of CEA and CA19-9 were 29.22 and 92.38, 14.67 and 94.55, respectively. **H**: The combined diagnosis of these two markers, sDC-SIGN and sDC-SIGNR, was analyzed through the binary logistic regression and ROC curve. The AUC of sDC-SIGN/sDC-SIGNR was 0.9885, the specificity and sensitivity were 94.8% and 98.7%, respectively.

According to our previous report, serum soluble LSECtin was detected at significantly higher level in colon cancer patients compared with the healthy controls [13], while the level of sDC-DIGNR in NHL was lower than that in the normal controls [23]. We therefore analyzed the levels of sDC-SIGN and sDC-SIGNR by ELISA. The sDC-SIGN level in the serum of patients with colon cancer (1.282±0.838 µg/ml) was significantly lower than that in healthy controls (2.687±1.178 µg/ml), P<0.05 (Fig. 1A). The sDC-SIGNR level was 594.90±595.54 ng/ml in colon cancer patients and 98.44±60.26 ng/ml in healthy controls. In contrast to the sDC-SIGN level, the sDC-SIGNR level in the colon cancer patients was higher than that in healthy controls, P<0.05 (Fig. 1B). DC-SIGN and DC-SIGNR, as the C-type lectins, were two related protein, and the levels of them may be correlated. So we analyzed the correlation between sDC-SIGN and sDC-SIGNR of the same patient. Interestingly, the level of sDC-SIGN was significantly correlated with that of sDC-SIGNR (r = 0.8173, P<0.0001) (Fig. 1C).

To assess the diagnostic value of sDC-SIGN and sDC-SIGNR serum levels in cancer patients, we generated ROC curves for these patients using GraphPad Prism5. Based on an analysis of the Youden index, the sensitivity and specificity for the best diagnostic concentration of sDC-SIGN (sDC-SIGN <2.226 µg/ml), which can differentiate colon cancer from tumor free individuals, were 87.56% and 55.56%, respectively (Fig. 1D). Moreover, the optimal concentration of sDC-SIGNR was more than 227.7 ng/ml, and the corresponding sensitivity and specificity were 61.90% and 97.50%, respectively (Fig. 1E). The AUC (area under the curve) of sDC-SIGN and sDC-SIGNR in patients was 0.7538 and 0.8031 respectively. Additionally, diagnosis of colon cancer with both sDC-SIGN and sDC-SIGNR was highly significant (P<0.0001). Meanwhile, CEA and CA19-9 are very important observational tumor markers used for clinical diagnosis and the determination of therapeutic efficacy in colon cancer. We also generated the ROC curves of CEA and CA19-9. According to the clinical decisive level (0–5 ug/l for CEA, 0–27 U/ml for CA19-9), we got the sensitivity and specificity for CEA and CA19-9 (Fig. 1F-1G). Compared with the AUC of sDC-SIGN (0.7538), sDC-SIGNR (0.8031), and CEA (0.7477), CA19-9 (0.6126) in patients was lower. What's more, the sensitivity of sDC-SIGN and sDC-SIGNR was 87.56 and 61.90 respectively, which is greatly higher than that of CEA (29.22) and CA19-9 (14.67). Additionally, when these two markers were combined to diagnose cancer patients, the specificity and sensitivity were 94.8% and 98.7%, respectively, and the AUC of DC-SIGN/DC-SIGNR was 0.9885 (Fig. 1H).

### The sDC-SIGN and sDC-SIGNR levels in colon cancer patients were not significantly correlated with CEA or CA19-9 levels, the degree of tumor cell differentiation, gender or age

To determine whether the levels of sDC-SIGN and sDC-SIGNR are different than that of CEA and CA19-9, we analyzed the correlation between the levels of sDC-SIGN or sDC-SIGNR in colon cancer patients with CEA and CA19-9, respectively. The results showed that the level of sDC-SIGN displayed no significant correlation with that of either CEA (29.271±83.517 µg/l, n = 171, r = −0.04) or CA19-9 (92.886±256.547 U/ml, n = 163, r = 0.103) nor was there significant correlation between the levels of sDC-SIGNR and CEA (r = −0.204, P>0.05) or CA19-9 (r = −0.004, P>0.05) (S2A–S2D Figure). This may indicate that these new markers can function as independent serum markers.

Next, we analyzed the correlation between the levels of sDC-SIGN or sDC-SIGNR and the following clinical data: gender and age. The sDC-SIGN and sDC-SIGNR levels in colon cancer patients showed no significant correlation with age (r = 0.019 and r = 0.029, S2E–S2F Figure), and there were no significant differences based on the gender of the patients (P>0.05, S2G–S2H Figure). This also indicates that these are independent biochemical indicators for colon cancer.

Additionally, the degree of differentiation of tumor cells is related to the speed of tumor growth, the degree of malignancy, the sensitivity to treatment and the overall prognosis. The samples were divided into the following five groups based on the degree of differentiation of tumor cells in the pathological results: well differentiated, moderately differentiated, poorly differentiated, well to moderately differentiated, and moderately to poorly differentiated. There was no significant difference between any two groups (S2I–S2J Figure), which implied that the levels of sDC-SIGN and sDC-SIGNR are not correlated with the degree of differentiation of colon cancer cells.

### The value of sDC-SIGN and sDC-SIGNR in the early diagnosis of stage I/II colon cancer patients

**Figure 2:**
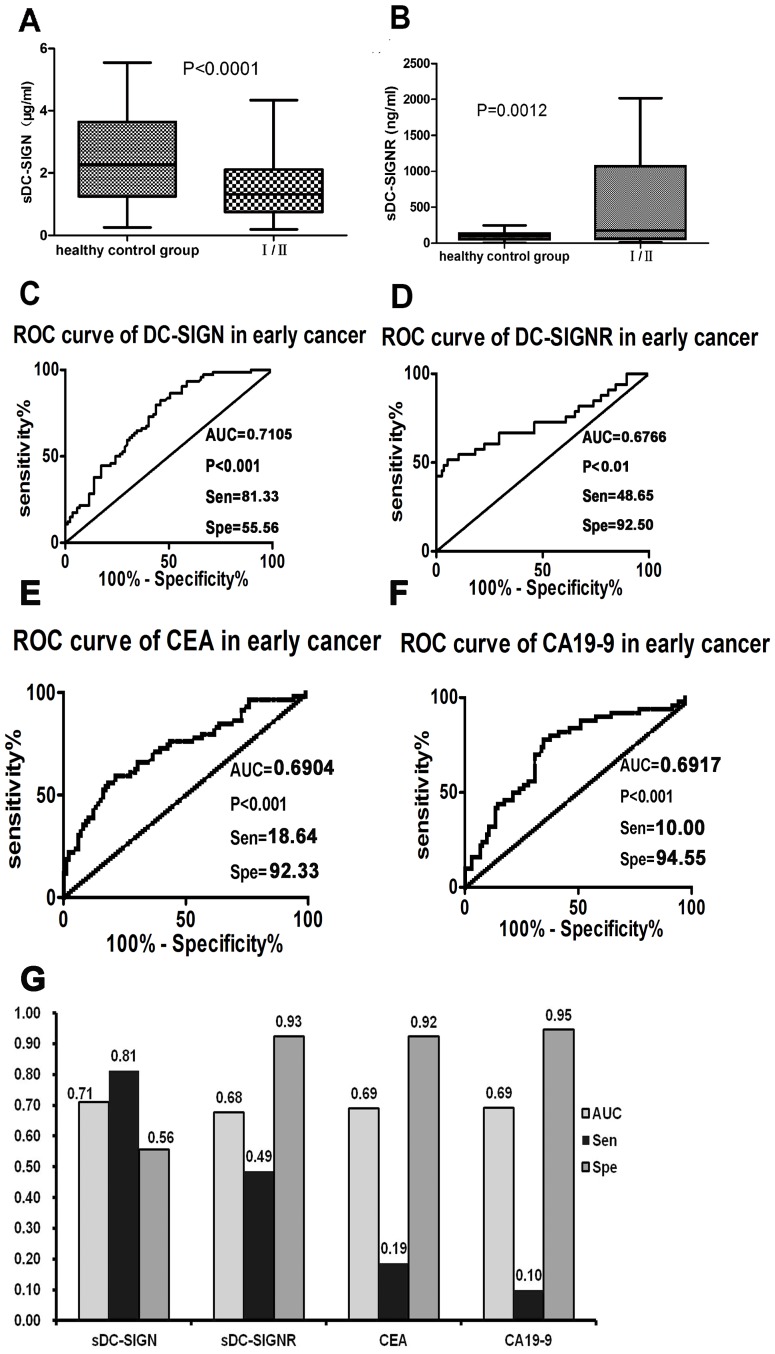
The early diagnostic values of sDC-SIGN and sDC-SIGNR in stage I/II colon cancer patients. **A–B**: Both sDC-SIGN and sDC-SIGNR levels from early colon cancer patients. Stage I/II patients were significantly different from healthy people, P<0.01. sDC-SIGN is lower than in the healthy control, while sDC-SIGNR is higher. **C–D**: In the stage I/II patients, sDC-SIGN and sDC-SIGNR had a significant diagnostic value (P<0.01). The cut-off concentrations of DC-SIGN and DC-SIGNR were less than 2.211 µg/ml and more than 189.3 ng/ml, respectively. The corresponding sensitivity and specificity of the two molecules were 81.33% and 55.56%, 48.65% and 92.50%, respectively. **E–F**: The AUC of CEA and CA19-9 in early cancer were 0.6904 and 0.6917. At the above clinical decisive level, the sensitivity of both CEA (18.64) and CA19-9 (10.00) were very low, while, the specificity of both CEA (92.33) and CA19-9 (94.55) was high. **G**: The comparison between the ROC curves of four markers, DC-SIGN, DC-SIGNR, CEA and CA19-9. There were significant differences between the AUCs of sDC-SIGN and both sDC-SIGNR and CA19-9. According to the cut-off values for sDC-SIGN and sDC-SIGNR obtained from the ROC curves, CEA and CA19-9 from the clinical decisive level, the sensitivity (diagnostic values) of both sDC-SIGN (81%) and sDC-SIGNR (49%) were higher than CEA (19%) and CA19-9 (10%).

According to a recent report, regenerating islet-derived protein 4 (REG4), a member of the C-type lectin superfamily, may be a good serum marker for the early diagnosis of gastric cancer [25]. We therefore analyzed the diagnostic values of sDC-SIGN and sDC-SIGNR in stage I/II colon cancer. Importantly, we found that the levels of sDC-SIGN and sDC-SIGNR in stage I/II cancer patients were 1.452±0.867 µg/ml and 505.5±645.1 ng/ml, respectively. These levels were significantly different than those in the healthy controls, P<0.05 (Fig. 2A–2B). In contrast to the higher level of sDC-SIGNR in early stage colon cancer patients relative to healthy controls, the level of sDC-SIGN in colon cancer was lower than in cancer-free people.

Next, the ROC curves of sDC-SIGN and sDC-SIGNR were generated to evaluate their diagnostic values in early stage colon cancer patients (Fig. 2C–2D). As in colon cancer at all stages, the diagnostic values of sDC-SIGN and sDC-SIGNR were both significantly effective at diagnosing stage I/II colon cancer (P<0.01). Moreover, the optimal concentrations of sDC-SIGN and sDC-SIGNR were less than 2.211 µg/ml and more than 189.3 ng/ml, respectively. Additionally, we also analyzed the diagnostic values of CEA, and CA19-9 in the diagnosis of early stage cancer patients (Fig. 2E–2F), the AUC, sensitivity and specificity of these four markers were showed in Fig. 2G. The sensitivity of sDC-SIGN were higher more than others markers (P<0.05), and the AUC of sDC-SIGN were significantly different from that of CA19-9 and sDC-SIGNR (P<0.05). For the proportion of abnormal expression of these four markers in early stage colon cancer patients, sDC-SIGN (81.3%) and sDC-SIGNR (40.5%) were higher than for CEA (21.3%) or CA19-9 (4.5%) (Table 1). On the whole, the levels of sDC-SIGN and sDC-SIGNR have early diagnostic potential for colon cancer patients.

**Table 1:** Clinical data of the colon cancer patients in ELISA study and the diagnostic values of different novel markers in the colon cancer with early stage.

Clinical data	DC-SIGN(n = 193)		DC-SIGNR(n = 84)	
	NO.	%	NO.	%
**Gender**				
Female	99	51.3	34	40.5
Male	94	48.7	50	59.5
**Age**				
≤60	83	43.0	34	40.5
>60	110	57.0	50	59.5
Median age (range)	61(21∼85)		62(33∼86)	
**TNM stage**	(n = 186)*		(n = 84)*	
I/II	73	39.2	37	44.0
III	44	23.7	21	25.0
IV	69	37.1	26	31.0
**Tumor differentiation**	(n = 161)*		(n = 66)*	
moderate	95	59	44	66.6
well	9	5.6	3	4.6
poor	6	3.8	5	7.6
well to moderate	23	14.2	8	12.1
moderate to poor	28	17.4	6	9.1
**CEA(n = 94)^#^**	**NO.**		**%**	
Normal	74		78.7	
High	20		21.3	
**CA199(n = 89)^#^**				
Normal	85		95.5	
High	4		4.5	
**DC-SIGN(n = 75)^#^**				
Normal	14		18.7	
Low	61		81.3	
**DC-SIGNR(n = 37)^#^**				
Normal	22		59.5	
High	15		40.5	

Note: “*” means that the samples are from all the colon cancer patients with surgical therapy; “**#**”means that the samples are from stage ??/?? colon cancer patients with surgical treatment.

### The prognostic values of sDC-SIGN and sDC-SIGNR in colon cancer patients

**Figure 3:**
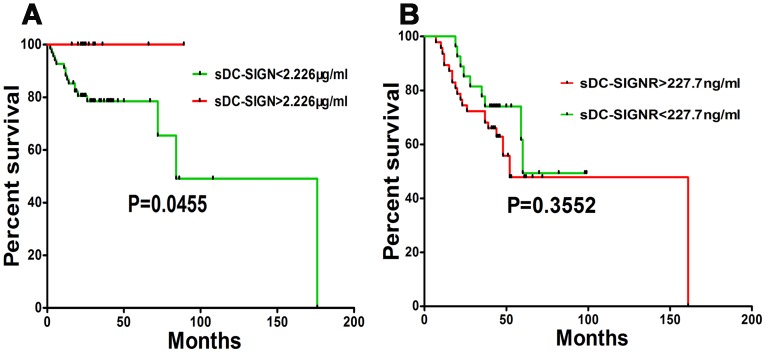
The prognostic significances of sDC-SIGN and sDC-SIGNR in cancer patients. The cut-off values of these markers were obtained from the above analysis of Youden index. **A**: There were significant differences in survival time between the patients (the level of sDC-SIGN>2.226 µg/ml) and the other patients (the level of sDC-SIGN>2.226 µg/ml) (P<0.05). **B**: the survival time of the patients (sDC-SIGNR<227.7) was not significantly different with that of the other patients (sDC-SIGNR>227.7) (P>0.05).

In order to find out whether the expression levels of sDC-SIGN and sDC-SIGNR in serum was correlated with the survival of patients, we generated the survival curves of sDC-SIGN and sDC-SIGNR through log-rank tests. According to the previous analysis of Youden index, we got the cut-off values of sDC-SIGN (2.226 µg/ml) and sDC-SIGNR (227.7 ng/ml) that differentiate the cancer patients from the tumor free individuals. Surprisingly, the survival time was significantly longer in the patients with higher levels of sDC-SIGN compared with the patients with lower levels of sDC-SIGN (P<0.05, Fig. 3A). However, there were not significant differences between the patients with high expression levels of sDC-SIGNR and those with low levels of sDC-SIGNR (P>0.05, Fig. 3B).

### IHC for DC-SIGN and DC-SIGNR expression in colon cancer tissues and in matched normal colonic mucosa of colon cancer patients

**Figure 4:**
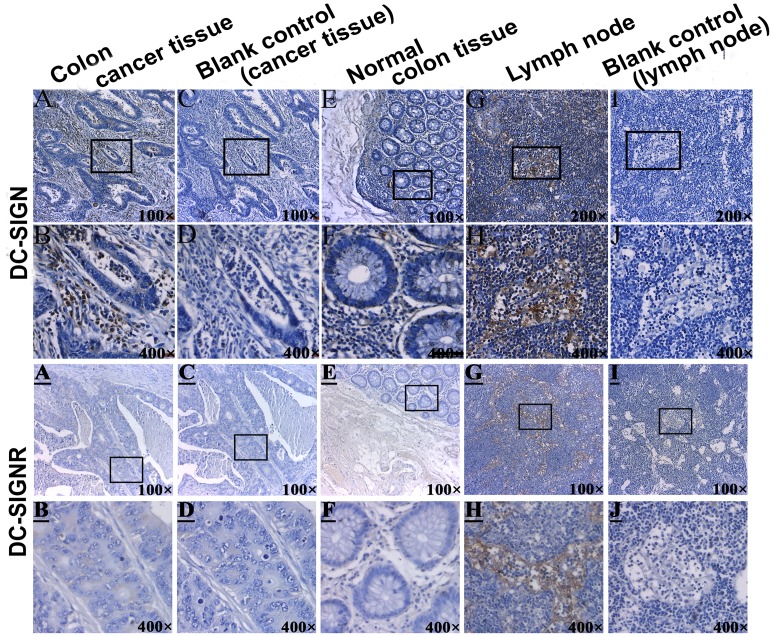
IHC for DC-SIGN and DC-SIGNR expression in colon cancer tissues and in matched normal colonic mucosa of colon cancer patients. Areas in the black boxes of **A**, **C**, **E**, **G**, **I** **and** **A**, **C**, **E**, **G** , **I** were enlarged below. **A-B**: DC-SIGN expression was detected in the internal border, central and peripheral part of colon carcinoma. **C–D**: blank control primary human colon cancer sections (without anti-DC-SIGN pAb). **E–F**: DC-SIGN expression in matched normal colonic mucosa. **G–H**: DC-SIGN expression was mainly detected in human lymphoid sinus. **I–J**: blank control human lymph node sections (without anti-DC-SIGN pAb); **A–B**: DC-SIGNR expression was weakly detected in the invasive margin of tumor. **C–D**: blank control primary human colon cancer sections. **E–F**: DC-SIGN expression was negative in matched normal colonic mucosa, while the **G** and **H** were positive controls in the human lymph node. **I–J**: blank control human lymph node sections. Magnification:100× in **A**, **C**, **E**,**A**, **C**, **E**, **G**,**I**; 200× in **G**, **I**; 400× in **B**, **D**, **F**, **H**, **J**, **B**, **D**, **F**, **H**, **J**.

We determined the expression level of both DC-SIGN and DC-SIGNR in serum. Moreover, it has been reported that immature DC-SIGN+ dendritic cells are present within primary colorectal cancer tissues [14], [26]. Our team previously found that DC-SIGNR is expressed in both cancer tissues and serum of NHL patients [23]. We therefore speculated that DC-SIGN and DC-SIGNR may be expressed in colon cancer tissues. IHC for DC-SIGN and DC-SIGNR was performed using 98 colon cancer tissues for DC-SIGN and 20 cancer tissues for DC-SIGNR, with the staining intensity determined by a pathologist who was blinded to the relevant clinical information. DC-SIGN staining in matched normal colonic mucosa was faint (Fig. 4E and 4F), while intense DC-SIGN staining was observed in the tumor stroma and the invasive margins of colon cancer tissues (Fig. 4A and 4B). The negative controls are shown in Fig. 4C and 4D. DC-SIGN staining in the lymph nodes, used as a positive control, are shown in Fig. 4G and 4H.

The results of the DC-SIGNR staining (Fig. 4A–J) showed that DC-SIGNR was negative in matched colonic tissues (Fig. 4E and 4F) and in the tumor stroma and was only weakly positive between the colon cancer foci (Fig. 4A and 4B). Therefore, DC-SIGNR expression in the colon cancer tissues was not analyzed further.

### Semi-quantitative image analysis of DC-SIGN expression in tissues and the analysis of its correlation with sDC-SIGN and CEA in serum from the same patient

**Figure 5:**
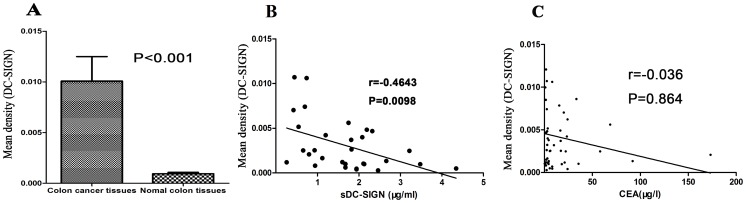
Semi-quantitative image analysis of DC-SIGN expression in tissues and the analysis of its correlation with sDC-SIGN and CEA in serum from the same patient. **A**: There was statistical significance in IHC for DC-SIGN expression between colon cancer tissues and matched normal colon tissues, P<0.001. DC-SIGN expression in colon cancer patients was higher than in normal colon tissues; **B**: The correlation between DC-SIGN staining intensity in colon cancer tissue and sDC-SIGN level in serum from the same patient. The mean density (Y axis) was negatively correlated with sDC-SIGN (X axis), r = −0.4643, P<0.01; **C**: The correlation between the mean density of DC-SIGN in colon cancer and CEA in serum. No significant correlation was observed between the mean density (Y axis) and CEA (X axis), with a Spearman correlation coefficient of -0.036, P>0.05.

As the expression of DC-SIGN was apparent within colon cancer tissues, we analyzed DC-SIGN immunostaining in both cancer tissues and matched normal colonic tissues by semi-quantitative image analysis. General information for the 49 deceased patients and 49 live patients are shown in Table 2. The mean density in matched colonic tissues was 0.0009391±0.0007490, and the upper 95% confidence level of the mean (cut-off value) was 0.001219. The mean density in colon cancer tissues (0.01009±0.02380) was higher than that in matched tissues (P<0.01, Fig. 5A).

**Table 2:** Clinical data and mean density of deceased colon cancer patients and live cancer patients in Immuunohistochemical study.

Clinical data	Dead patients(n = 49)		Live patients(n = 49)	
	NO.	%	NO.	%
**Gender**				
Female	17	34.7	20	40.8
Male	32	65.3	29	59.2
Age				
≤60	2	4.1	24	49.0
>60	47	95.9	25	51.0
Median age (range)	73(49∼88)		60(28∼78)	
**TNM stage**				
I/II	16	32.7	22	44.9
III	17	34.6	21	42.9
IV	16	32.7	6	12.2
**Mean density**				
>0.001219	34	69.4	33	67.3
<0.001219	15	30.6	16	32.7
**Median survival time (range)**	14(0∼94)months		—	

From the above results, we found that the high level of DC-SIGN expression in colon cancer tissue was reversed in serum, which displayed a low level of sDC-SIGN. We collected 30 serum samples from preoperative colon cancer patients and analyzed the correlation between DC-SIGN staining intensity in colon cancer tissue and the level of sDC-SIGN in serum from the same patient. As shown in Fig. 5B, DC-SIGN expression in colon cancer tissue had a significantly negative correlation with the sDC-SIGN level in serum (r = −0.4643, P<0.01).

As DC-SIGN was reported to show a high affinity for Le glycans on CEA [14]-[15], we also analyzed the correlation between DC-SIGN expression in colon cancer tissue and the level of CEA in serum from the same patient. The results showed no significant correlation (r = −0.036, P>0.05, Fig. 5C).

### Aberrant correlation between DC-SIGN expression in colon cancer tissues and patient survival

**Figure 6:**
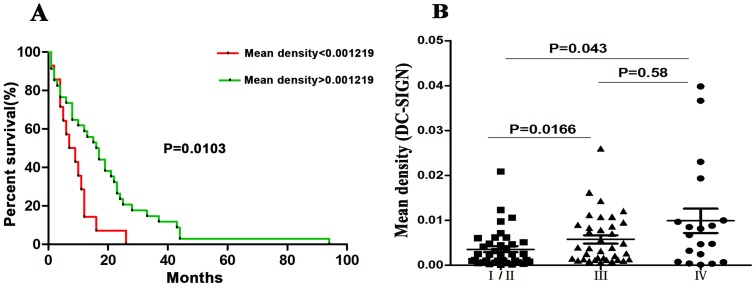
Aberrant correlation between DC-SIGN expression in colon cancer tissues and patient survival. **A**: Survival curves by DC-SIGN expression in colon cancer patients. The percent survival of patients with a mean density>0.001219 was higher than for those with mean density <0.001219 (Mantel-Cox, P = 0.009). **B**: The mean density of DC-SIGN in stage I/II colon cancer patients were notably lower than that in III or IV colon cancer patients according to TNM staging system, P<0.05.

The specific interaction between Le glycans and DC-SIGN [15](Nonaka et al., 2008), the novel function of DC-SIGN in establishing the initial contact between DCs and resting T cells [27]–[28], and the infiltration of DCs into primary colorectal cancer have all been found to be associated with patient survival of and tumor progression [29]–[30]. Moreover, REG4, a member of the C-type lectin superfamily, was reported to be a potential prognostic indicator for the evaluation of the survival time of gastric cancer patients [25]. We speculated that there might be a correlation between DC-SIGN expression level in colon cancer tissues and patient survival. Therefore, samples from 49 deceased patients (shown in S4 Table) were divided into two different patient groups using a cut-off value of 0.001219, and the percent survival was then analyzed using log-rank tests. To our surprise, the percent survival of patients with a mean density>0.001219 was higher than for those with a low mean density (Mantel-Cox, P = 0.009, Fig. 6A).

Next, we analyzed the expression levels of DC-SIGN in cancer tissues at different stages. The mean density of DC-SIGN in stage I/II colon cancer patients (0.003551±0.004211, n = 38) was significantly lower than that in either stage III colon cancer patients (0.005747±0.005559, n = 36) or stage IV colon cancer patients (0.009898±0.01175, n = 19), P<0.05 (Fig. 6B).

## Discussion

Based on known characteristics of tumor growth, a rough calculation suggests that it may take several years for a cancerous cell to form a tumor with approximately 10^9^ cells that could be detected by the clinical imaging tests [31]. However, early tumor tissue containing approximately 10^6^ cells may secret tumor markers found in the serum. Therefore, serum marker tests are very important for the early diagnosis of cancer, especially for the screening of high-risk populations, as these tests are simple and practical compared with imaging tests. If cancers are detected at their earliest stages, or even in the premalignant state, physicians will have a higher probability of treating and truly curing these cancers [32]–[33]. Recently, the desire to find molecular markers that have high sensitivity and specificity in detecting early CRC has been increasing.

In the present study, we determined the levels of sDC-SIGN and sDC-SIGNR in serum from patients with colon cancer and found that the levels of sDC-SIGN were significantly correlated with that of sDC-SIGNR. Moreover, the levels in colon cancer patients were significantly different from those in healthy people, but with differing trends in the changes for each maker. Additionally, the levels of these markers in serum from early stage colon cancer patients were significantly different from the healthy controls. Surprisingly, the sensitivity of sDC-SIGN and sDC-SIGNR were greatly higher than that of widely-used markers, CEA and CA19-9, in cancer with all stage or early stage, which is good for colon cancer screening and diagnosis. Moreover, combined diagnosis of these two markers, sDC-SIGN and sDC-SIGNR, had high specificity (94.8%), sensitivity (98.7%) and AUC (0.9885). Meanwhile, high level of sDC-SIGN was accompanied with the long survival time. Therefore, we suggest that sDC-SIGN may be useful for colon cancer screening and prognosis, and sDC-SIGNR should be applied for diagnosis of early stage colon cancer. The combined diagnosis of them was better than both of them alone. The detection of sDC-SIGN and sDC-SIGNR in serum leads to an obvious question: in what manner is DC-SIGN secreted? One potential mechanism is the direct regulation of mRNA expression; another potential mechanism is the conditional cleavage of the extracellular portion of the membrane-bound protein. Mummidi reported the potential existence of sDC-SIGN variants at the cDNA level, generated by alternative splicing of the exon encoding the transmembrane domain. This may lead to the expression of the sDC-SIGN protein [34]. In addition, Martinez identified an sDC-SIGN isoform that lacked the putative transmembrane domain at the cDNA and protein level. When this sDC-SIGN cDNA was transfected into the CHO cells, sDC-SIGN was detected in the cytoplasm but not in the culture supernatants of immature and stimulated DCs. However, it was also found that sDC-SIGN protein expressed from this cDNA was nonsecreted and nonfunctional and could not participate in the activation of T cells [35]. Later, Plazolles found that the failure to detect sDC-SIGN in culture supernatants by Martinez may have been the result of a lack of sensitivity in their ELISA assays as well as failure to concentrate the culture supernatant before measuring sDC-SIGN [36]. Their study showed that sDC-SIGN, which was secreted in the course of DC differentiation, was not expressed as an exosome-associated protein but as a full length variant. Furthermore, their sDC-SIGN was functional and promoted CMV infection of MoDC. Based on the research above, as well as our own results, we speculate that DC-SIGN is released from DCs during the process of DC maturation and flows into the serum via a number of physiological and pathological processes. There is no relevant research pertaining to the mechanism of DC-SIGNR secretion. However, according to our results, the level of sDC-SIGNR was significantly correlated with that of sDC-SIGN in cancer patients. Moreover, these two molecules were homogenous. Therefore, we speculated that DC-SIGNR may have the similar way as DC-SIGN to secret although they were expressed in different cells. Additionally, both of them were immunological molecules, DC-SIGNR and DC-SIGN may be involved in similar immune activities in cancer patients. Whether our speculation is correct or not, further research is needed to verify this phenomenon.

To determine whether sDC-SIGN and sDC-SIGNR are independent biological tumor markers, we analyzed their correlation with some important clinical factors for the diagnosis and prognosis of CRC, including CEA and CA19-9 levels, age, gender, and the degree of differentiation of tumor cells. There was no relationship between sDC-SIGN and sDC-SIGNR and any of these factors, indicating that sDC-SIGN and sDC-SIGNR are most likely independent serum markers. Many studies have reported higher levels of Lewis antigen, resulting from the abnormal glycosylation of CEA on colon epithelial cells, which has specific affinity for DC-SIGN (but not for DC-SIGNR) [37]–[38]. Since, in our experiment, the CEA level was not correlated with sDC-SIGN in serum (similar to the results regarding the expression of DC-SIGN of colon cancer tissues), we speculate that the expression of either factor has no effect on the other. One possible reason is that CEA in serum from CRC patients is secreted by the colon cancer cells themselves, with a small amount being shed directly into the blood. The level of CEA will increase until the cancer cells invade a vein or lymph-vessel [4], [39]. Another possible explanation is the presence of other ligands for DC-SIGN on colon epithelial cells. Nonaka reported that Mac-2BP, a protein expressed on some colon carcinoma cells, could be recognized by DC-SIGN through Le glycans [26]. Recently this group also found that tumor-associated Lewis glycans displayed affinity for another C-type serum lectin, mannan-binding protein, with fructose being involved in this interaction rather than mannose [40]. Therefore the interaction between DC-SIGN and colon cancer cells may not simply be one-for-one, which is an idea that is consistent with our experimental data.

In our immunohistochemical experiments, we found that DC-SIGN was more highly expressed in colon cancer tissue compared with normal colon tissue, which is a result that was contradictory to our results regarding the sDC-SIGN levels in serum. This was confirmed by an analysis of the correlation between the expression intensity and the level of sDC-SIGN from the same patient, two values that were negatively correlated (r = −0.4643). This interesting result gives us a hint regarding the manner in which DC-SIGN is secreted, suggesting that it might be released by DCs during the processes of maturation. This is supported by the following: DC-SIGN was highly expressed in immature DCs and poorly expressed in mature DCs [18]. While DC-SIGN expressed in immature DCs interacts with carcinoembryonic antigen (CEA) expressed on colorectal carcinoma cells, this interaction might impair the functional maturation and differentiation of immature DCs [14]–[15]. Therefore, if a cancer patient displays high DC-SIGN expression in tumor tissue, immature DCs may be inhibited, and the release of DC-SIGN would be blocked. Surprisingly, we found that high-intensity DC-SIGN staining in colon cancer patients correlated with longer patient survival, which contradicted the results from advanced-stage patients with higher DC-SIGN expression (Fig. 6B), as the late TNM stage was often associated with worse prognosis [41]. However, others have reported that the relationship between the TNM stage and prognosis is not obvious, except for stage IV[42]. We therefore analyzed the relationship between survival and TNM stage in our collected patients and observed that only the survival of stage IV patients was shorter (data not shown). Meanwhile, when we removed the stage IV patients from our survival analysis, high DC-SIGN expression was still associated with longer patient survival. Our results are potentially consistent with previous reports relating the infiltration of colon cancer DCs with patient prognosis. Patients with a high number of immature DCs (S100 positive) had a longer survival [30], [43]–[44], and DC-SIGN was highly expressed on immature DCs in local cancer tissues. Therefore, we have demonstrated that the DC-SIGN expression level in colon cancer tissues may have prognostic value for colon cancer patients.

In conclusion, DC-SIGN and DC-SIGNR may be used as independent markers for the early detection of colon cancer and for evaluating patient prognosis. It is also worth noting that although DC-SIGN and DC-SIGNR are homologous, they display differing levels of expression and differing trends in the changes to those levels between cancer tissues and serum. They therefore play different roles in the progression and prognosis of colon cancer. Many questions remain unanswered that will require many *in vivo* and *in vitro* experiments to be properly addressed. Ultimately, these future experiments will most likely confirm DC-SIGN and DC-SIGNR as useful biological markers for the diagnosis and prognosis of colon cancer.

## Supporting Information

S1 Figure The Standard Curve of sDC-SIGN and sDC-SIGNR. **A-B**: Standard Curve fitting of rhDC-SIGN (**A**) and rhDC-SIGNR (**B**), Linear regression was completed successfully, R^2^ = 0.9987 and R^2^ = 0.9971, respectively.
(TIF)

S2 Figure The sDC-SIGN and sDC-SIGNR levels in colon cancer patients were not significantly correlated with CEA, CA19-9, and so on. Both sDC-SIGN and sDC-SIGNR levels (Y axis) were not significantly correlated with CEA (**A–B**) or CA19-9 (X axis) (**C–D**) levels based on a Spearman correlation coefficient, P>0.05. **E–F**: No significant correlation was observed between the sDC-SIGN (Y axis) or sDC-SIGNR (Y axis) levels and age (X axis), with Spearman correlation coefficients of 0.019 or 0.029, respectively, P>0.05. **G–H**: Scatter plots of sDC-SIGN and sDC-SIGNR levels in patients of different gender. There was no significant difference between male and female patients, P>0.05. **I–J**: Comparison of the levels of sDC-SIGN and sDC-SIGNR in five groups according to the degree of tumor cells' differentiation; each dot represents the sDC-SIGN or sDC-SIGNR level for one patient. There were no significant differences between any two groups, P>0.05.
(TIF)

S1 Table Clinical data of the colon cancer patients in DC-SIGN ELISA study.
(DOC)

S2 Table Clinical data of the colon cancer patients in DC-SIGNR ELISA study.
(DOC)

S3 Table Clinical data of the colon cancer patients whose serum were collected in immunohistochemical study.
(DOC)

S4 Table Clinical data of the colon cancer patients whose serum were collected in immunohistochemical study.
(DOC)
