# Supplementary figures and images for: The Clinical Significance of DC-SIGN and DC-SIGNR, which Are Novel Markers Expressed in Human Colon Cancer
Source: PLoS One. 2014 Dec 12;9(12):e114748. doi: 10.1371/journal.pone.0114748 (PMC4264775; doi:10.1371/journal.pone.0114748)

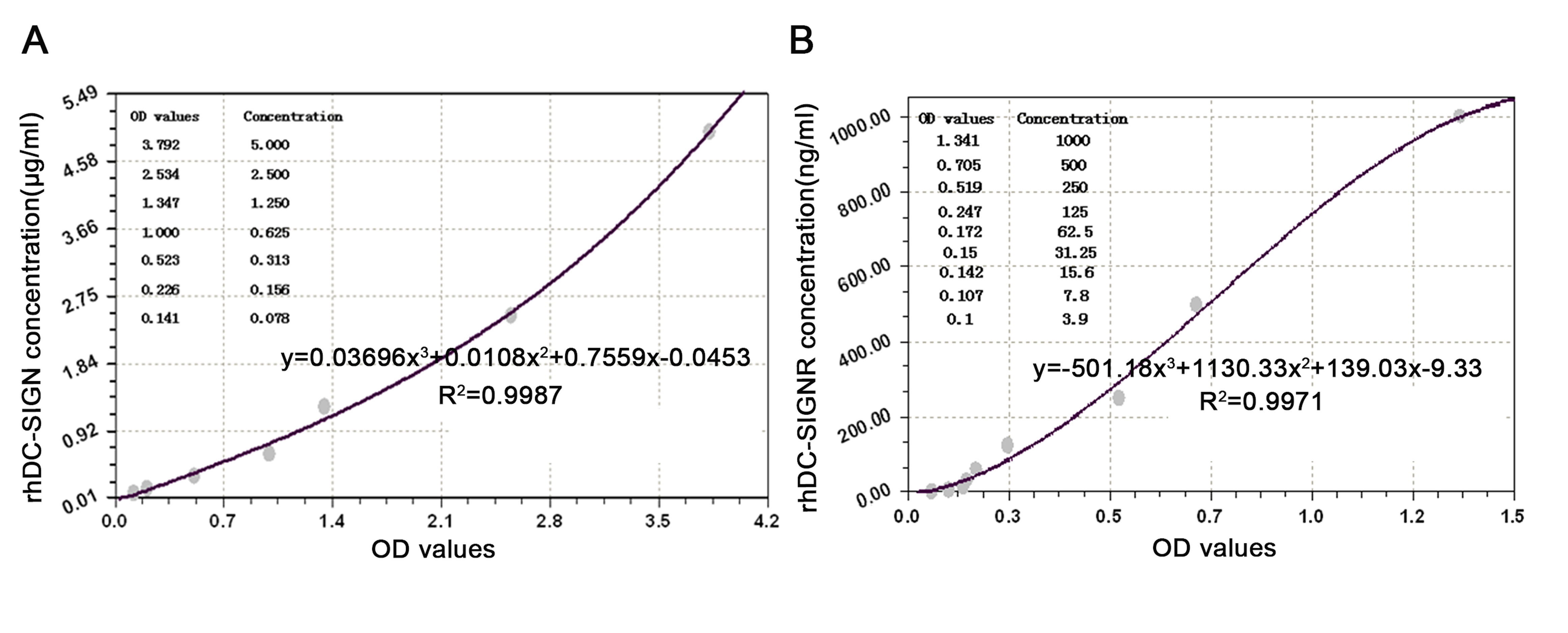

Supplement: S1 Figure — The Standard Curve of sDC-SIGN and sDC-SIGNR. A-B: Standard Curve fitting of rhDC-SIGN (A) and rhDC-SIGNR (B), Linear regression was completed successfully, R2 = 0.9987 and R2 = 0.9971, respectively. (TIF) [file pone.0114748.s001.tif]

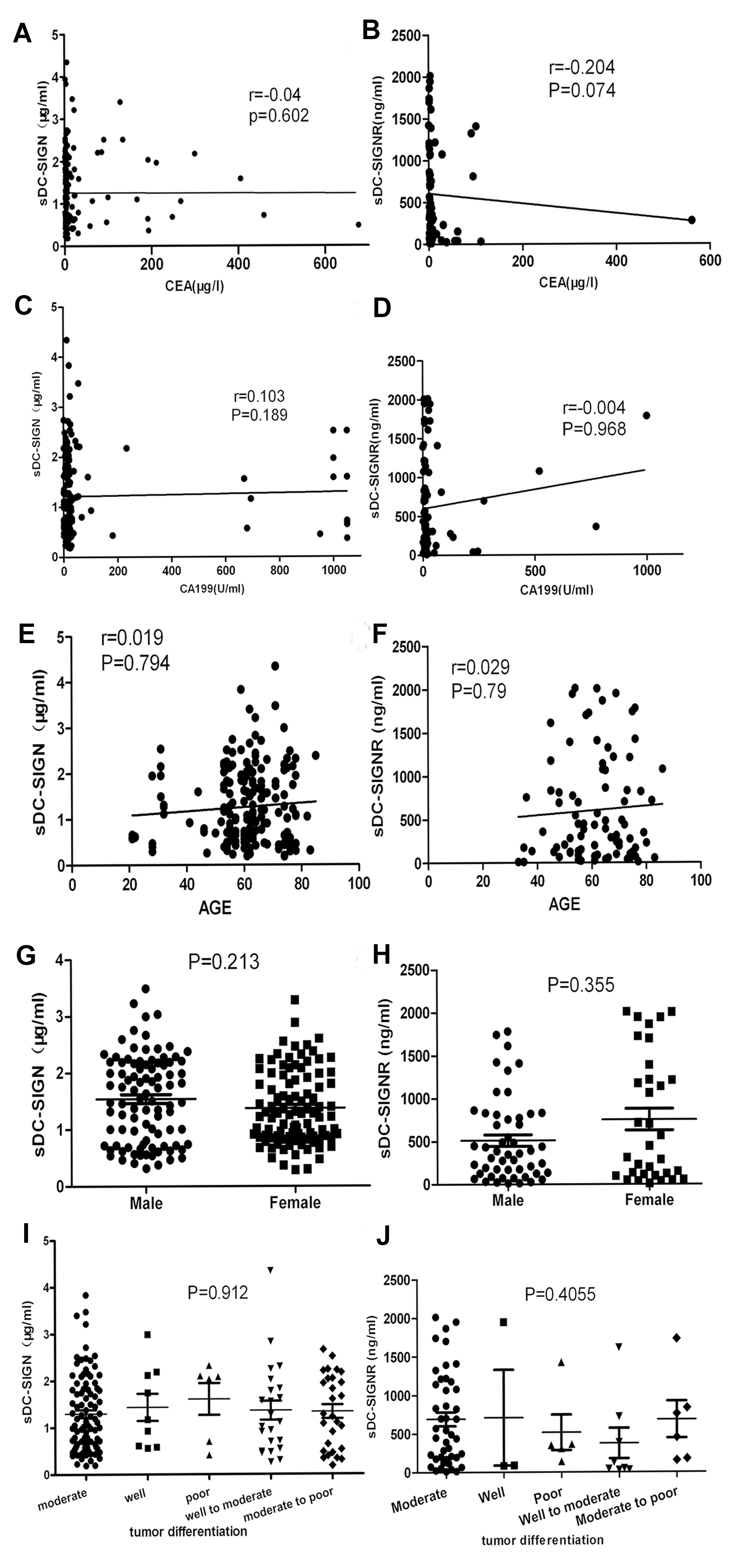

Supplement: S2 Figure — The sDC-SIGN and sDC-SIGNR levels in colon cancer patients were not significantly correlated with CEA, CA19-9, and so on. Both sDC-SIGN and sDC-SIGNR levels (Y axis) were not significantly correlated with CEA (A–B) or CA19-9 (X axis) (C–D) levels based on a Spearman correlation coefficient, P>0.05. E–F: No significant correlation was observed between the sDC-SIGN (Y axis) or sDC-SIGNR (Y axis) levels and age (X axis), with Spearman correlation coefficients of 0.019 or 0.029, respectively, P>0.05. G–H: Scatter plots of sDC-SIGN and sDC-SIGNR levels in patients of different gender. There was no significant difference between male and female patients, P>0.05. I–J: Comparison of the levels of sDC-SIGN and sDC-SIGNR in five groups according to the degree of tumor cells' differentiation; each dot represents the sDC-SIGN or sDC-SIGNR level for one patient. There were no significant differences between any two groups, P>0.05. (TIF) [file pone.0114748.s002.tif]
