# Supplementary material for: The Clinical Significance of DC-SIGN and DC-SIGNR, which Are Novel Markers Expressed in Human Colon Cancer
Source: PLoS One. 2014 Dec 12;9(12):e114748. doi: 10.1371/journal.pone.0114748 (PMC4264775; doi:10.1371/journal.pone.0114748)
Supplement: S1 Table — Clinical data of the colon cancer patients in DC-SIGN ELISA study. (DOC) [file pone.0114748.s003.doc]

Table S1 Clinical data of the colon cancer patients in DC-SIGN ELISA study

| No. | Gender/age | Tumor stage | Tumor differentiation | sDC-SIGN | CEA (μg/l) | CA199 (U/ml) | Survival time |
| --- | --- | --- | --- | --- | --- | --- | --- |
|  |
| 1 | F/74 | Ⅲ | Moderate to poor | 0.185 | 5.59 | 23.72 | 12* |
| 2 | F/61 | Ⅱ | Moderate | 0.193 | 6.97 | 18.37 | 46 |
| 3 | M/74 | Ⅳ | Moderate | 0.201 | - | - | - |
| 4 | F/56 | Ⅲ | Moderate | 0.23 | 2.89 | 27.95 | 39 |
| 5 | M/62 | - | - | 0.238 | 1.63 | 5.92 | 22 |
| 6 | M/47 | Ⅲ | Well to moderate | 0.259 | 3.84 | 14.53 | - |
| 7 | M/28 | Ⅲ | - | 0.297 | 5.94 | 12.88 | - |
| 8 | M/76 | Ⅱ | Moderate | 0.298 | 3 | 12.67 | - |
| 9 | F/78 | Ⅱ | Well to moderate | 0.305 | 30.7 | 14.95 | 26* |
| 10 | M/54 | Ⅳ | Moderate to poor | 0.314 | 3.62 | 7.59 | - |
| 11 | F/83 | - | - | 0.315 | 1.95 | 13.35 | 108 |
| 12 | F/72 | Ⅲ | Moderate to poor | 0.342 | 2.01 | - | - |
| 13 | M/54 | Ⅳ | Moderate to poor | 0.342 | 4.04 | 8.62 | - |
| 14 | M/76 | Ⅱ | Moderate | 0.343 | 3.3 | 11.61 | - |
| 15 | M/76 | Ⅱ | Moderate | 0.352 | - | - | 26 |
| 16 | F/76 | Ⅲ | - | 0.354 | 4.29 | 17.21 | - |
| 17 | M/62 | - | - | 0.36 | - | - | - |
| 18 | M/60 | Ⅳ | - | 0.364 | 193 | >1050 | - |
| 19 | M/53 | Ⅲ | Moderate to poor | 0.392 | 2.79 | 10.85 | 37 |
| 20 | M/28 | Ⅲ | - | 0.4 | 5.81 | 10.89 | - |
| 21 | M/54 | Ⅲ | Moderate | 0.411 | 3.65 | 7.09 | - |
| 22 | M/61 | Ⅳ | Poor | 0.413 | 17.44 | 27.25 | 18 |
| 23 | M/54 | Ⅱ | - | 0.42 | 2.57 | 11.86 | - |
| 24 | F/64 | Ⅲ | Moderate | 0.425 | 20.11 | 0.965 | 19 |
| 25 | F/57 | Ⅱ | Moderate | 0.425 | 1.27 | 18.36 | - |
| 26 | M/72 | Ⅲ | - | 0.433 | 16.33 | 181.3 | 5* |
| 27 | F/74 | Ⅱ | Moderate | 0.436 | 2.79 | - | 26 |
| 28 | F/74 | Ⅳ | - | 0.442 | >850 | >950 | 2* |
| 29 | M/68 | Ⅲ | Moderate | 0.447 | 2.39 | 13.43 | 30 |
| 30 | F/75 | Ⅱ | Moderate | 0.447 | 3.2 | 9.86 | 86 |
| 31 | M/54 | Ⅳ | Moderate to poor | 0.456 | 2.95 | 8.13 | - |
| 32 | F/78 | Ⅱ | Well to moderate | 0.459 | - | - | - |
| 33 | M/28 | Ⅲ | - | 0.471 | 4.06 | 9.65 | 26 |
| 34 | M/54 | Ⅱ | - | 0.473 | 3.26 | 12.29 | 67 |
| 35 | M/74 | Ⅳ | Moderate | 0.476 | 58.06 | 35.78 | 6* |
| 36 | F/67 | Ⅳ | Well to moderate | 0.479 | 676.9 | <0.57 | 31 |
| 37 | M/54 | Ⅳ | Moderate to poor | 0.48 | 2.73 | 7.04 | 20* |
| 38 | F/76 | Ⅲ | Moderate | 0.482 | 1.46 | 12.71 | - |
| 39 | F/76 | Ⅲ | Moderate | 0.489 | 2.42 | 12.43 | - |
| 40 | M/50 | Ⅲ | Well to moderate | 0.49 | - | - | - |
| 41 | M/54 | Ⅳ | - | 0.495 | 4.1 | 30.97 | 25 |
| 42 | F/57 | Ⅱ | Moderate | 0.513 | 1.44 | 14.73 | - |
| 43 | F/55 | Ⅱ | Moderate to poor | 0.532 | 3.1 | 17.97 | - |
| 44 | F/61 | Ⅱ | Moderate | 0.544 | 4.84 | 11.19 | - |
| 45 | M/75 | Ⅰ | Well to moderate | 0.547 | 3.43 | 12.6 | 22 |
| 46 | F/61 | Ⅳ | Well | 0.562 | 2.75 | 23.76 | - |
| 47 | F/60 | Ⅲ | Moderate | 0.563 | 1.73 | 13.75 | 42 |
| 48 | F/63 | Ⅳ | - | 0.565 | 96.21 | 679 | - |
| 49 | F/21 | Ⅳ | Moderate | 0.581 | 9.47 | <0.601 | - |
| 50 | F/61 | Ⅳ | Well | 0.587 | 2.39 | 18.03 | 42 |
| 51 | F/76 | Ⅲ | Moderate | 0.591 | 2.89 | 12.72 | 31 |
| 52 | F/21 | Ⅳ | Moderate | 0.593 | 6.42 | <0.600 | - |
| 53 | F/62 | Ⅳ | Moderate | 0.598 | 2.34 | 6.49 | - |
| 54 | F/21 | Ⅳ | Moderate | 0.609 | 9.31 | <0.600 | - |
| 55 | F/21 | Ⅳ | Moderate | 0.611 | 19.9 | 0.797 | - |
| 56 | F/61 | Ⅳ | Well | 0.613 | 2.55 | 20.53 | - |
| 57 | F/21 | Ⅳ | Moderate | 0.625 | 18.75 | <0.600 | - |
| 58 | F/63 | Ⅳ | - | 0.64 | 191.2 | >1000 | - |
| 59 | F/74 | Ⅳ | Moderate to poor | 0.644 | 23.45 | 18.9 | 12* |
| 60 | F/62 | Ⅳ | Moderate | 0.644 | 4.11 | 9.84 | - |
| 61 | M/60 | Ⅳ | - | 0.653 | 2.63 | 17.25 | - |
| 62 | F/57 | Ⅱ | Moderate | 0.656 | 1.62 | 20.51 | 24 |
| 63 | F/21 | Ⅳ | Moderate | 0.659 | 15.83 | <0.600 | 50 |
| 64 | F/63 | Ⅳ | - | 0.683 | 247.8 | >1000 | - |
| 65 | M/50 | Ⅲ | Well to moderate | 0.697 | 2.4 | 7.6 | 17 |
| 66 | F/75 | Ⅲ | Poor | 0.702 | 8.83 | 20.24 | - |
| 67 | M/54 | Ⅲ | Moderate | 0.71 | 3.63 | 6.15 | 43 |
| 68 | F/55 | Ⅲ | Moderate | 0.714 | 11.74 | 24.42 | - |
| 69 | F/63 | Ⅳ | - | 0.719 | 458.9 | 1050 | 84* |
| 70 | M/46 | - | - | 0.719 | 1.64 | 5.82 | - |
| 71 | M/60 | Ⅲ | Well to moderate | 0.72 | 3.1 | 0.68 | 67 |
| 72 | M/60 | Ⅱ | Moderate | 0.723 | 4.83 | 30.31 | 26 |
| 73 | M/60 | Ⅳ | - | 0.73 | 5.33 | 26.93 | 30 |
| 74 | F/57 | Ⅱ | Moderate | 0.734 | 1.18 | 18.7 | - |
| 75 | F/46 | Ⅱ | Moderate | 0.74 | 8.01 | 6.83 | 23 |
| 76 | F/64 | Ⅲ | Moderate | 0.749 | 3.18 | 0.51 | - |
| 77 | F/66 | Ⅳ | Moderate | 0.771 | 4.72 | 29.7 | 72* |
| 78 | F/46 | Ⅱ | Moderate | 0.795 | 0.724 | 7.52 | - |
| 79 | F/55 | Ⅲ | Moderate | 0.799 | 31.18 | 66.18 | 19 |
| 80 | M/62 | Ⅲ | Moderate | 0.807 | 2.02 | 24.87 | 72 |
| 81 | F/55 | Ⅱ | Moderate to poor | 0.903 | 3.37 | 21.06 | - |
| 82 | M/67 | Ⅱ | Moderate | 0.907 | - | - | - |
| 83 | F/68 | Ⅱ | Well to moderate | 0.91 | - | - | - |
| 84 | F/41 | Ⅱ | Moderate | 0.929 | 2.34 | 27.25 | 30 |
| 85 | F/64 | Ⅱ | Well | 0.933 | 17.02 | 101.3 | - |
| 86 | M/52 | Ⅱ | Well to moderate | 0.944 | 2.44 | 18.74 | 30 |
| 87 | F/64 | Ⅲ | Moderate | 0.956 | 3.65 | - | - |
| 88 | M/63 | Ⅱ | Moderate | 0.97 | 3.08 | 20.72 | 22 |
| 89 | F/57 | Ⅲ | Moderate | 0.985 | 3.72 | 4.44 | 40 |
| 90 | F/63 | Ⅲ | Moderate | 0.992 | 4.28 | 0.63 | 39 |
| 91 | F/61 | - | - | 0.995 | 3.91 | 26.7 | 40 |
| 92 | F/62 | Ⅳ | Moderate | 1.009 | 5.76 | 13.7 | 35 |
| 93 | M/75 | Ⅰ | Well to moderate | 1.02 | - | - | - |
| 94 | M/75 | Ⅳ | Moderate to poor | 1.022 | 2.17 | 5.33 | 41 |
| 95 | F/57 | Ⅲ | Moderate | 1.026 | 4.06 | 6.25 | - |
| 96 | M/66 | Ⅳ | Moderate | 1.049 | 2.63 | 12.12 | - |
| 97 | M/82 | Ⅱ | Moderate | 1.056 | 267.2 | 0.613 | 17 |
| 98 | F/78 | Ⅳ | - | 1.067 | 63.65 | 5.89 | 26 |
| 99 | M/61 | Ⅱ | Moderate to poor | 1.083 | - | - | - |
| 100 | F/78 | Ⅳ | - | 1.099 | 166.7 | 7.24 | - |
| 101 | F/55 | Ⅱ | Moderate to poor | 1.108 | 3.29 | 24.14 | 30 |
| 102 | F/63 | Ⅲ | Well to moderate | 1.119 | 1.76 | 5.85 | 23 |
| 103 | F/32 | Ⅱ | Moderate | 1.119 | 1.77 | <0.63 | 29 |
| 104 | M/72 | Ⅲ | Moderate | 1.14 | 5.74 | 29.96 | 35 |
| 105 | F/63 | Ⅳ | - | 1.157 | 99.65 | 693.5 | - |
| 106 | M/53 | Ⅳ | Well | 1.173 | 1.5 | 25.67 | - |
| 107 | M/66 | Ⅳ | Moderate | 1.196 | 22.18 | 45.37 | - |
| 108 | M/67 | Ⅱ | Moderate | 1.2 | 23.35 | 23.54 | 17 |
| 109 | F/77 | Ⅳ | - | 1.219 | 4.06 | 54.33 | 11* |
| 110 | M/54 | Ⅳ | Moderate to poor | 1.225 | 2.88 | 24.74 | - |
| 111 | F/53 | Ⅳ | Moderate | 1.247 | 2.91 | - | 36 |
| 112 | M/74 | Ⅱ | Well to moderate | 1.276 | 5.67 | 3.01 | 3* |
| 113 | F/32 | Ⅱ | Moderate | 1.279 | 2.54 | <0.64 | - |
| 114 | F/55 | Ⅱ | Moderate to poor | 1.28 | 3.82 | 18.23 | - |
| 115 | F/63 | Ⅲ | Moderate | 1.307 | 2.67 | 0.813 | - |
| 116 | F/32 | Ⅱ | Moderate | 1.316 | 1.87 | <0.63 | - |
| 117 | F/56 | Ⅱ | - | 1.356 | 5.45 | 1.09 | - |
| 118 | M/60 | Ⅱ | Moderate | 1.384 | 3.18 |  | - |
| 119 | M/66 | Ⅳ | Moderate | 1.453 | 8.75 | 11.98 | - |
| 120 | F/71 | Ⅱ | Moderate | 1.456 | 3.61 | 21.46 | - |
| 121 | F/31 | Ⅳ | Moderate | 1.496 | 1.61 | 23.67 | 42 |
| 122 | M/62 | Ⅲ | Moderate | 1.503 | - | - | - |
| 123 | M/74 | Ⅳ | Moderate | 1.529 | - | - | - |
| 124 | M/57 | Ⅱ | Well to moderate | 1.556 | 2.9 | 668.8 | 176* |
| 125 | M/67 | Ⅱ | Moderate | 1.559 | 4.18 |  | - |
| 126 | F/68 | Ⅱ | Well to moderate | 1.574 | 2 | 14.09 | - |
| 127 | F/63 | Ⅳ | - | 1.584 | 405.2 | >1000 | - |
| 128 | F/61 | Ⅳ | - | 1.592 | 32.34 | 1050 | - |
| 129 | M/44 | Ⅱ | Moderate to poor | 1.602 | 4.03 | 89.8 | - |
| 130 | M/60 | Ⅲ | Well to moderate | 1.625 | 3.04 | 0.639 | - |
| 131 | M/66 | Ⅳ | Moderate | 1.635 | 15.72 | 13.67 | - |
| 132 | F/53 | Ⅱ | Moderate to poor | 1.659 | 2.29 | 20.93 | - |
| 133 | M/64 | Ⅱ | Moderate | 1.675 | 4.27 | 5.34 | - |
| 134 | M/66 | Ⅳ | Moderate | 1.721 | 11.8 | 37 | - |
| 135 | F/71 | Ⅱ | Moderate | 1.727 | 4.2 | 19.6 | - |
| 136 | M/53 | Ⅳ | Well | 1.745 | 1.87 | 20.75 | - |
| 137 | M/77 | Ⅲ | Moderate | 1.749 | - | - | 43 |
| 138 | F/63 | Ⅲ | Well to moderate | 1.781 | 2.47 | 5.4 | - |
| 139 | M/55 | Ⅱ | Moderate | 1.801 | 1.59 | 8.63 | - |
| 140 | F/71 | Ⅱ | Moderate | 1.815 | 4.08 | 17.14 | 25 |
| 141 | M/66 | Ⅳ | Moderate | 1.817 | 22.29 | 14.09 | - |
| 142 | M/60 | Ⅲ | Moderate to poor | 1.821 | 2.34 | 11.54 | 14* |
| 143 | F/63 | Ⅲ | Well to moderate | 1.833 | 2.23 | 7.56 | - |
| 144 | M/54 | Ⅱ | Moderate | 1.837 | 1.16 | 12.45 | - |
| 145 | F/59 | Ⅳ | Moderate | 1.849 | - | - | - |
| 146 | M/61 | Ⅱ | Moderate to poor | 1.942 | 8.08 | 20.59 | 18 |
| 147 | M/78 | Ⅱ | Moderate | 1.949 | 2.35 | 13.16 | - |
| 148 | F/28 | Ⅳ | Moderate to poor | 1.95 | 2.48 | - | 29 |
| 149 | F/31 | Ⅳ | Moderate | 1.956 | 1.63 | 20.29 | - |
| 150 | F/59 | Ⅳ | Moderate | 1.964 | 211.5 | >1000 | 13* |
| 151 | F/63 | Ⅲ | Well to moderate | 2.024 | 1.95 | 5.44 | - |
| 152 | F/53 | Ⅳ | Moderate to poor | 2.033 | 192.2 | 18.62 | 18* |
| 153 | M/62 | Ⅲ | Poor | 2.034 | 1.41 | 11.11 | - |
| 154 | M/61 | Ⅱ | Moderate to poor | 2.045 | - | - | - |
| 155 | M/74 | Ⅳ | - | 2.07 | 4.91 | 14.75 | - |
| 156 | M/62 | Ⅲ | Poor | 2.082 | 3.31 | 16.69 | - |
| 157 | M/66 | Ⅳ | Moderate | 2.101 | 11 | 15.04 | - |
| 158 | M/62 | Ⅲ | Poor | 2.102 | - | - | - |
| 159 | M/54 | Ⅱ | Moderate | 2.108 | 0.278 | 11.27 | - |
| 160 | F/75 | Ⅱ | Well | 2.118 | 1.63 | 9.24 | 30 |
| 161 | M/54 | Ⅱ | Moderate | 2.125 | 1.27 | 12.33 | - |
| 162 | M/55 | Ⅱ | Moderate | 2.13 | 1.24 | 8.55 | 28 |
| 163 | M/77 | Ⅲ | Moderate | 2.156 | 5.71 | 11.6 | 31 |
| 164 | F/31 | Ⅳ | Moderate | 2.157 | 1.49 | 22.31 | - |
| 165 | F/53 | Ⅳ | Moderate to poor | 2.176 | 299.8 | 233.8 | - |
| 166 | F/56 | Ⅱ | - | 2.18 | 4.07 | <0.63 | 36 |
| 167 | M/74 | Ⅰ | Well | 2.191 | 2.78 | 10.49 | - |
| 168 | F/53 | Ⅳ | Moderate to poor | 2.208 | 76.19 | 58.62 | - |
| 169 | F/53 | Ⅳ | Moderate to poor | 2.221 | 85.1 | 49.41 | - |
| 170 | M/61 | Ⅱ | Moderate to poor | 2.235 | - | - | - |
| 171 | M/64 | Ⅱ | Well to moderate | 2.248 | - | - | 24 |
| 172 | M/54 | Ⅱ | Moderate | 2.251 | 1.27 | 12.33 | 30 |
| 173 | F/68 | Ⅱ | Well to moderate | 2.304 | 2.08 | 13.37 | 30 |
| 174 | M/78 | Ⅱ | Moderate | 2.321 | 2.42 | - | 25 |
| 175 | F/75 | Ⅲ | Poor | 2.324 | 21.02 | 43.88 | 18* |
| 176 | M/69 | Ⅳ | - | 2.381 | 6.71 | 7.94 | - |
| 177 | F/64 | Ⅱ | Moderate | 2.383 | - | - | - |
| 178 | M/64 | Ⅱ | Moderate | 2.442 | - | - | - |
| 179 | M/56 | Ⅱ | Moderate | 2.462 | 2.22 | 29.4 | 24 |
| 180 | M/76 | Ⅳ | Moderate | 2.488 | 1.43 | 4.35 | 66 |
| 181 | M/54 | Ⅳ | Moderate to poor | 2.515 | 134 | >1050 | - |
| 182 | F/59 | Ⅳ | Moderate | 2.515 | 90.27 | >1000 | - |
| 183 | F/31 | Ⅳ | Moderate | 2.541 | 1.76 | 24.11 | - |
| 184 | F/62 | Ⅲ | Moderate to poor | 2.66 | 3.79 | 24.84 | 22 |
| 185 | M/66 | Ⅳ | Moderate | 2.719 | 8.34 | 16.62 | 31 |
| 186 | F/56 | Ⅱ | - | 2.742 | 5.37 | <0.64 | 4* |
| 187 | M/64 | Ⅱ | Well to moderate | 2.83 | - | - | - |
| 188 | M/74 | Ⅰ | Well | 2.993 | - | - | 27 |
| 189 | F/64 | Ⅱ | Moderate | 3.218 | 22.2 | 24.1 | 23 |
| 190 | M/62 | Ⅳ | Moderate | 3.402 | 128.3 | - | 89 |
| 191 | M/71 | Ⅳ | Moderate | 3.478 | 18.05 | 55.22 | 16 |
| 192 | M/59 | Ⅱ | Moderate | 3.837 | 3.51 | 20.08 | 22 |
| 193 | M/71 | Ⅰ | Well to moderate | 4.344 | 5.43 | 12.66 | 20 |

Note: F: female; M: male; CEA, carcinoembryonic antigen; CA199, carbohydrate antigen 199; -: not available; *: Death/Event.
