# Supplementary material for: The Clinical Significance of DC-SIGN and DC-SIGNR, which Are Novel Markers Expressed in Human Colon Cancer
Source: PLoS One. 2014 Dec 12;9(12):e114748. doi: 10.1371/journal.pone.0114748 (PMC4264775; doi:10.1371/journal.pone.0114748)
Supplement: S2 Table — Clinical data of the colon cancer patients in DC-SIGNR ELISA study. (DOC) [file pone.0114748.s004.doc]

Table S2 Clinical data of the colon cancer patients in DC-SIGNR ELISA study

| No. | Gender/age | Tumor stage | Tumor differentiation | sDC-SIGNR | CEA | CA199 | Survival time |
| --- | --- | --- | --- | --- | --- | --- | --- |
|  |  |  |  | (ng/ml) | (μg/l) | (U/ml) |  |
| 1 | M/35 | Ⅰ | - | 12.376 | 5.2 | 16.22 | 37* |
| 2 | F/72 | Ⅲ | moderate | 703.461 | 3.54 | 9.51 | 46 |
| 3 | F/53 | Ⅳ | well | 1947.793 | 3.45 | 32.88 | 12* |
| 4 | M/45 | Ⅱ | Well to moderate | 1613.842 | 5.05 | 23.4 | 26* |
| 5 | M/48 | Ⅲ | Moderate | 693.665 | 2.11 | 4.53 | 43 |
| 6 | F/80 | Ⅱ | Moderate | 229.988 | 2.24 | 4.3 | 39 |
| 7 | F/74 | Ⅱ | - | 100.529 | - | - | 22* |
| 8 | M/78 | Ⅳ | Moderate | 820.942 | 2.1 | 13.55 | 7* |
| 9 | M/64 | Ⅳ | - | 1076.591 | 28.62 | 520.62 | 161* |
| 10 | F/38 | Ⅲ | Poor | 136.265 | 0.9 | 6.09 | 98 |
| 11 | M/75 | Ⅳ | - | 124.812 | 16.29 | 58.51 | 24* |
| 12 | M/76 | Ⅳ | - | 1781.686 | >1000 | >1000 | 23* |
| 13 | M/45 | Ⅲ | Moderate | 831.327 | 1.71 | 4.59 | 72 |
| 14 | M/61 | Ⅳ | - | 433.961 | 5.66 | 7.02 | 17* |
| 15 | F/61 | Ⅱ | Moderate | 200.651 | 2.32 | 8.96 | 50 |
| 16 | M/82 | Ⅰ | Well to moderate | 31.693 | 110.7 | 49.49 | 41 |
| 17 | F/82 | Ⅲ | Well to moderate | 718.019 | 1.84 | 4.92 | 41 |
| 18 | F/33 | Ⅱ | Moderate | 11.213 | 2.85 | - | 42 |
| 19 | F/65 | Ⅱ | Moderate | 1066.014 | 2.59 | 26.03 | 39 |
| 20 | F/68 | Ⅱ | Moderate | 1220.12 | 13.43 | 5.03 | 52* |
| 21 | M/65 | Ⅲ | Moderate | 391.959 | 2.83 | 12.51 | 46 |
| 22 | M/53 | Ⅲ | - | 774.411 | 2.64 | 20.08 | 41 |
| 23 | F/73 | Ⅰ | Moderate | 41.116 | 60.71 | 17.2 | 40 |
| 24 | F/67 | Ⅳ | - | 290.137 | 6.23 | 13.11 | 44* |
| 25 | F/55 | Ⅲ | Moderate to poor | 448.728 | 0.98 | 10.43 | 45 |
| 26 | F/69 | Ⅱ | Moderate | 1951.285 | 2.92 | 7.37 | 61 |
| 27 | M/76 | Ⅲ | poor | 1423.172 | 0.2 | 3.94 | - |
| 28 | M/86 | Ⅱ | Moderate | 1078.271 | 2.71 | 2.77 | - |
| 29 | M/47 | Ⅱ | Moderate | 176.294 | 3.51 | 9.61 | 41 |
| 30 | M/50 | Ⅱ | Moderate | 208.758 | 2.57 | 17.64 | 99 |
| 31 | M/83 | Ⅰ | Moderate | 55.286 | 2.1 | 13.55 | 43 |
| 32 | F/74 | Ⅱ | Moderate | 1212.695 | 0.96 | 9.69 | 44 |
| 33 | M/61 | Ⅰ | - | 74.243 | 0.81 | 6.4 | - |
| 34 | F/74 | Ⅰ | Well to moderate | 49.456 | 1.72 | 8.38 | 60* |
| 35 | M/57 | Ⅲ | Moderate | 448.225 | 1.42 | 6.42 | 41 |
| 36 | F/59 | Ⅲ | Moderate to poor | 1728.886 | 1.57 | 32.93 | 53 |
| 37 | M/75 | Ⅳ | Moderate | 1741.966 | 1.01 | 7.86 | 44 |
| 38 | F/64 | Ⅱ | Moderate | 1142.515 | 1.7 | 12.59 | 62 |
| 39 | M/69 | Ⅱ | Moderate | 243.058 | - | - | 44 |
| 40 | M/79 | Ⅲ | Poor | 347.632 | 1.6 | 7.36 | 44 |
| 41 | M/76 | Ⅱ | Moderate | 71.638 | 0.59 | 7.3 | 53 |
| 42 | F/69 | Ⅱ | - | 99.481 | 2.09 | 10.36 | 48* |
| 43 | F/64 | Ⅱ | Moderate | 1867.958 | 1.38 | 26.78 | 44 |
| 44 | M/55 | Ⅲ | Well | 85.343 | 2.88 | 6.79 | 44 |
| 45 | F/52 | Ⅱ | Moderate | 125.298 | 1.14 | 4.8 | 70 |
| 46 | F/62 | Ⅳ | - | 2006.629 | 890.9 | 6.1 | 19* |
| 47 | M/65 | Ⅲ | - | 484.616 | 3.41 | 19.7 | 46 |
| 48 | M/77 | Ⅰ | Moderate | 12.64 | 1.55 | 18.06 | 82 |
| 49 | M/48 | Ⅳ | - | 810.672 | 94.47 | 82.31 | - |
| 50 | M/61 | Ⅱ | Well to moderate | 25.002 | 37.65 | 0.65 | 35* |
| 51 | F/74 | Ⅳ | - | 49.456 | 26.46 | 244.3 | 20* |
| 52 | F/65 | Ⅳ | Well to moderate | 41.002 | 54.78 | 222.7 | 45 |
| 53 | F/45 | Ⅲ | Moderate | 1178.015 | 0.78 | 8.07 | 42 |
| 54 | M/70 | Ⅱ | Moderate | 194.362 | - | - | - |
| 55 | M/71 | Ⅲ | Moderate | 489.685 | 3.64 | 22.62 | 66 |
| 56 | M/70 | Ⅳ | - | 231.398 | 30.44 | 134.5 | - |
| 57 | M/46 | Ⅱ | Well to moderate | 125.136 | 2.78 | 11.58 | 46 |
| 58 | M/42 | Ⅳ | Poor | 360.309 | 6.58 | 773 | 17* |
| 59 | M/55 | Ⅳ | Moderate | 696.676 | >1000 | 272.6 | 10* |
| 60 | F/62 | Ⅳ | Moderate | 571.789 | 1.3 | 8.82 | 15* |
| 61 | F/66 | Ⅳ | - | 57.398 | 4.17 | 13.46 | 19* |
| 62 | M/72 | Ⅱ | Moderate | 439.533 | 2.35 | <0.6 | - |
| 63 | F/54 | Ⅱ | Moderate | 2014.168 | 3.47 | 22.9 | 45 |
| 64 | M/52 | Ⅲ | Moderate | 283.906 | 6.85 | 20.22 | - |
| 65 | F/58 | Ⅱ | Moderate | 1700.65 | 1.19 | 8.06 | 46 |
| 66 | M/74 | Ⅳ | - | 276.862 | 561.3 | 122.3 | 12* |
| 67 | M/35 | Ⅳ | Moderate to poor | 176.477 | 7.72 | 2.73 | 59* |
| 68 | M/54 | Ⅱ | Moderate | 547.05 | 1.87 | 4.09 | 43 |
| 69 | F/69 | Ⅲ | Moderate | 314.774 | 0.66 | 21.87 | 11* |
| 70 | M/36 | Ⅳ | Moderate to poor | 758.014 | 5.1 | 6.84 | 37* |
| 71 | M/62 | Ⅳ | Moderate | 1409.646 | 100.9 | 64.78 | 22* |
| 72 | M/56 | Ⅳ | Poor | 306.363 | 9.31 | 42.47 | 51 |
| 73 | M/62 | Ⅲ | Well | 89.637 | 3.59 | 9.95 | 42 |
| 74 | M/65 | Ⅲ | Moderate | 864.251 | 1.3 | 8.87 | 20* |
| 75 | F/52 | Ⅳ | Moderate | 1392.96 | 5.02 | <0.6 | 37* |
| 76 | F/52 | Ⅳ | Moderate | 115.131 | 3.31 | 15.8 | - |
| 77 | M/73 | Ⅳ | Moderate to poor | 831.716 | 2.94 | - | 43 |
| 78 | M/57 | Ⅱ | Well to moderate | 385.624 | 2.06 | 6.98 | 43 |
| 79 | M/55 | Ⅱ | - | 134.267 | - | - | - |
| 80 | M/76 | Ⅲ | Moderate | 170.594 | 4.46 | <0.6 | 28* |
| 81 | F/56 | Ⅱ | Moderate to poor | 152.796 | 61.3 | 21.16 | 41 |
| 82 | F/56 | Ⅱ | Moderate | 23.841 | 3.6 | 7.46 | 41 |
| 83 | M/66 | Ⅳ | Moderate | 1326.547 | 90.96 | - | 39* |
| 84 | M/48 | Ⅱ | Moderate | 65.042 | 7.21 | 13.45 | 41 |

Note: F: female; M: male; CEA, carcinoembryonic antigen; CA199, carbohydrate antigen 199; -: not available; *: Death/Event.
