# Supplementary material for: The Clinical Significance of DC-SIGN and DC-SIGNR, which Are Novel Markers Expressed in Human Colon Cancer
Source: PLoS One. 2014 Dec 12;9(12):e114748. doi: 10.1371/journal.pone.0114748 (PMC4264775; doi:10.1371/journal.pone.0114748)
Supplement: S3 Table — Clinical data of the colon cancer patients whose serum were collected in immunohistochemical study. (DOC) [file pone.0114748.s005.doc]

Table S3 Clinical data of the colon cancer patients whose serum were collected in immunohistochemical study

| No. | Gender/age | Tumor stage | Tumor differentiation | CEA (μg/l) | CA199 (μ/ml) | sDC-SIGN (μg/ml) | Mean density |
| --- | --- | --- | --- | --- | --- | --- | --- |
| 1 | M/77 | Ⅲ | Moderate | 68.53 | 48.42 | 1.749 | 0.0056036 |
| 2 | M/68 | Ⅲ | Moderate | 2.39 | 13.43 | 0.447 | 0.0107071 |
| 3 | M/54 | Ⅱ | Moderate | 0.278 | 11.27 | 2.108 | 0.0010341 |
| 4 | M/74 | Ⅳ | Moderate | 58.06 | 35.78 | 0.476 | 0.0024687 |
| 5 | F/61 | Ⅱ | Well | 20.99 | 60.36 | - | 0.0011654 |
| 6 | M/54 | Ⅳ | Moderate to poor | 2.24 | 4.3 | - | 0.0047577 |
| 7 | F/55 | Ⅱ | Moderate to poor | 24.09 | 221.9 | - | 0.0062149 |
| 8 | F/56 | Ⅲ | Moderate | 4.48 | 21.46 | - | 0.0013335 |
| 9 | M/54 | Ⅲ | Moderate | 4.34 |  | - | 0.0037785 |
| 10 | M/55 | Ⅱ | Moderate | 0.833 | 0.872 | 2.130 | 0.0009863 |
| 11 | M/74 | Ⅰ | Well | 2.78 | 10.49 | 2.191 | 0.0048337 |
| 12 | F/74 | Ⅲ | Moderate to poor | 33.27 | 13.72 | - | 0.0086084 |
| 13 | M/53 | Ⅲ | Moderate to poor | 16.61 | 67.16 | - | 0.0049115 |
| 14 | F/57 | Ⅲ | Moderate | 1.6 | 2.95 | - | 0.0120606 |
| 15 | F/53 | Ⅲ | Moderate | 9.86 | 5.74 | - | 0.0031929 |
| 16 | M/60 | Ⅲ | Well to moderate | 3.92 |  | - | 0.0010602 |
| 17 | M/60 | Ⅲ | Well to moderate | 6.02 | 184 | - | 0.0025364 |
| 18 | M/56 | Ⅱ | Moderate | 2.22 | 29.4 | 2.462 | 0.0002739 |
| 19 | M/28 | Ⅲ | - | 15.39 | 11.26 | - | 0.00784 |
| 20 | F/64 | Ⅱ | Moderate | 22.2 | 24.1 | 3.218 | 0.0024705 |
| 21 | F/46 | Ⅱ | Moderate | 8.01 | 6.83 | 0.740 | 0.0106062 |
| 22 | F/71 | Ⅱ | Moderate | 16.82 | 20.12 | 1.815 | 0.0036977 |
| 23 | F/61 | Ⅱ | Moderate | 35.43 | 15.85 | - | 0.0010147 |
| 24 | F/75 | Ⅱ | Moderate | 7.99 |  | - | 0.0006947 |
| 25 | M/78 | Ⅱ | Moderate | 1.75 | 11.77 | 2.321 | 0.0046756 |
| 26 | F/64 | Ⅱ | Well | 17.02 | 101.3 | 0.933 | 0.0025355 |
| 27 | M/75 | Ⅰ | Well to moderate | 3.43 | 12.6 | 0.547 | 0.0051557 |
| 28 | F/78 | Ⅳ | - | 24.04 | 1.66 | - | 0.0004053 |
| 29 | M/75 | Ⅳ | Moderate to poor | 1.41 | 4.54 | - | 0.0085081 |
| 30 | M/54 | Ⅳ | Moderate | 2.91 | 17.74 | - | 0.0032288 |
| 31 | F/57 | Ⅱ | Moderate | 5.51 | 16.86 | 0.656 | 0.0025109 |
| 32 | F/31 | Ⅲ | Moderate | 1.76 | 160.1 | - | 0.0014899 |
| 33 | F/63 | Ⅲ | Well to moderate | 3.04 | 48.83 | 1.119 | 0.0016497 |
| 34 | M/47 | Ⅲ | Well to moderate | 3.84 | 14.53 | 0.259 | 0.0011802 |
| 35 | M/71 | Ⅰ | Well to moderate | 5.43 | 12.66 | 4.344 | 0.0004942 |
| 36 | F/62 | Ⅲ | Moderate to poor | 92.01 | >950 | 2.660 | 0.0013277 |
| 37 | F/64 | Ⅲ | Moderate | 20.11 | 0.965 | 0.425 | 0.0070239 |
| 38 | M/61 | Ⅱ | Moderate to poor | 8.08 | 20.59 | 1.942 | 0.0004607 |
| 39 | F/55 | Ⅲ | Moderate | 173.2 | 274.1 | 0.799 | 0.0020715 |
| 40 | M/76 | Ⅳ | Moderate | 0.84 | 2.4 | - | 0.0099682 |
| 41 | M/62 | Ⅲ | Poor | 3.31 | 16.69 | 2.082 | 0.0039903 |
| 42 | M/52 | Ⅱ | Well to moderate | 1.52 | 9.9 | 0.944 | 0.0008054 |
| 43 | M/64 | Ⅱ | Moderate | 4.27 | 5.34 | 1.675 | 0.0009922 |
| 44 | F/41 | Ⅱ | Moderate | 1.48 | 8.36 | - | 0.0011943 |
| 45 | M/44 | Ⅱ | Moderate to poor | 4.03 | 89.8 | 1.602 | 0.0012056 |
| 46 | M/67 | Ⅱ | Moderate | 23.35 | 23.54 | 1.200 | 0.0042109 |
| 47 | M/50 | Ⅲ | Well to moderate | 2.4 | 7.6 | 0.697 | 0.0074091 |
| 48 | M/60 | Ⅲ | Moderate to poor | 2.34 | 11.54 | 1.821 | 0.0026237 |
| 49 | M/71 | Ⅲ | Moderate | 18.05 | 55.22 | 3.478 | 0.0009732 |

Note: F: female; M: male; CEA, carcinoembryonic antigen; CA199, carbohydrate antigen 199; -: not available.
