# Supplementary material for: The Clinical Significance of DC-SIGN and DC-SIGNR, which Are Novel Markers Expressed in Human Colon Cancer
Source: PLoS One. 2014 Dec 12;9(12):e114748. doi: 10.1371/journal.pone.0114748 (PMC4264775; doi:10.1371/journal.pone.0114748)
Supplement: S4 Table — Clinical data of the colon cancer patients whose serum were collected in immunohistochemical study. (DOC) [file pone.0114748.s006.doc]

Table S4 Clinical data of the dead colon cancer patients in immunohistochemical study

Note: F: female; M: male; -: not available.

| N0. | Gender/age | Tumor stage | Tumor differentiation | First visit | Date of death | Mean density |
| --- | --- | --- | --- | --- | --- | --- |
| 1 | F/88 | Ⅱ | Moderate | 201205 | 201206 | 0.0060525 |
| 2 | M/80 | Ⅳ | Moderate to poor | 201109 | 201111 | 0.0081362 |
| 3 | F/76 | Ⅲ | Well to moderate | 201201 | 201201 | 0.0095124 |
| 4 | F/62 | Ⅲ | Moderate | 201003 | 201110 | 0.0089995 |
| 5 | M/79 | Ⅱ | Moderate | 201104 | 201104 | 0.0023116 |
| 6 | F/65 | Ⅱ | Moderate | 201102 | 201102 | 0.0005801 |
| 7 | F/83 | Ⅲ | Moderate | 201010 | 201011 | 0.0003259 |
| 8 | F/66 | Ⅱ | - | 201102 | 201108 | 0.0044772 |
| 9 | M/74 | Ⅳ | Moderate | 201105 | 201205 | 0.023017 |
| 10 | F/74 | Ⅱ | Moderate | 201001 | 201203 | 0.0010249 |
| 11 | M/60 | Ⅳ | Moderate to poor | 200912 | 201102 | 0.0047001 |
| 12 | M/78 | Ⅲ | Well to moderate | 200907 | 201007 | 0.0009118 |
| 13 | M/78 | Ⅱ | Well to moderate | 201104 | 201204 | 0.0006574 |
| 14 | M/77 | Ⅳ | Moderate | 201004 | 201109 | 0.0003262 |
| 15 | F/79 | Ⅱ | Well to moderate | 200805 | 200805 | 0.0001215 |
| 16 | F/79 | Ⅱ | Moderate | 200805 | 200805 | 0.003171 |
| 17 | M/76 | Ⅱ | Moderate | 200802 | 200803 | 0.0003705 |
| 18 | F/74 | Ⅲ | Poor | 200802 | 200810 | 0.0142888 |
| 19 | M/54 | Ⅲ | Poor | 200801 | 200911 | 0.0016316 |
| 20 | M/71 | Ⅲ | Moderate | 200712 | 200911 | 0.0012234 |
| 21 | M/61 | Ⅲ | Well | 200707 | 200806 | 0.0006271 |
| 22 | M/75 | Ⅳ | Moderate | 200707 | 200811 | 0.0067205 |
| 23 | M/79 | Ⅱ | Moderate | 201108 | 201110 | 0.0123149 |
| 24 | F/73 | Ⅳ | Poor | 200605 | 200701 | 0.1489637 |
| 25 | M/81 | Ⅲ | Moderate | 200801 | 200810 | 0.0011786 |
| 26 | M/75 | Ⅲ | Moderate | 200612 | 201001 | 0.0107691 |
| 27 | M/76 | Ⅱ | Moderate | 200602 | 200606 | 0.0062115 |
| 28 | M/66 | Ⅱ | Moderate | 200612 | 200612 | 0.0208731 |
| 29 | M/70 | Ⅲ | Moderate | 200511 | 200706 | 0.0039015 |
| 30 | M/76 | Ⅱ | Moderate | 200508 | 200510 | 0.0071394 |
| 31 | F/71 | Ⅳ | Moderate | 200505 | 200505 | 0.0398036 |
| 32 | M/73 | Ⅲ | Poor | 200503 | 200509 | 0.0007976 |
| 33 | M/75 | Ⅳ | Moderate | 200501 | 200605 | 0.0087827 |
| 34 | M/76 | Ⅳ | Moderate | 200607 | 201003 | 0.0193255 |
| 35 | M/77 | Ⅳ | Poor | 200912 | 201004 | 0.000131 |
| 36 | F/64 | Ⅲ | Moderate | 200404 | 200801 | 0.0161945 |
| 37 | F/83 | Ⅲ | Moderate | 200312 | 200511 | 0.0111613 |
| 38 | M/76 | Ⅲ | Moderate | 200604 | 200604 | 0.0083448 |
| 39 | M/78 | Ⅱ | Moderate | 200305 | 200410 | 0.0023621 |
| 40 | M/78 | Ⅳ | Poor | 200304 | 200309 | 0.0007942 |
| 41 | M/75 | Ⅳ | Moderate | 200302 | 200407 | 0.0569607 |
| 42 | F/79 | Ⅰ | Moderate | 200302 | 200511 | 0.009782 |
| 43 | M/60 | Ⅳ | - | 200301 | 200502 | 0.0592061 |
| 44 | M/80 | Ⅱ | Moderate | 200806 | 200903 | 0.0002515 |
| 45 | F/75 | Ⅲ | Moderate | 200211 | 200410 | 0.0259694 |
| 46 | M/75 | Ⅳ | Poor | 200303 | 200312 | 0.0366253 |
| 47 | F/66 | Ⅳ | Moderate | 201102 | 201107 | 0.0007003 |
| 48 | M/71 | Ⅲ | Moderate | 200301 | 201011 | 0.165289 |
| 49 | M/70 | Ⅳ | Poor | 201110 | 201202 | 0.0096572 |
